# Functional role of small extrachromosomal circular DNA in colorectal cancer

**DOI:** 10.1073/pnas.2523047123

**Published:** 2026-04-02

**Authors:** Judith Mary Hariprakash, Egija Zole, Weijia Feng, Dan Hao, Lasse Bøllehuus Hansen, Nirmalya Bandyopadhyay, Marghoob Mohiyuddin, Sihan Wu, Astrid Zedlitz Johansen, Julia Sidenius Johansen, Birgitte Regenberg

**Affiliations:** ^a^Ecology and Evolution, Department of Biology, University of Copenhagen, Copenhagen 2100, Denmark; ^b^Bioinformatics Research & Early Development, Roche Diagnostics, Santa Clara, CA 95050; ^c^Children’s Medical Center Research Institute, University of Texas Southwestern Medical Center, Dallas, TX 75235; ^d^Department of Pathology, Copenhagen University Hospital—Herlev and Gentofte, Herlev 2730, Denmark; ^e^Department of Oncology, Copenhagen University Hospital—Herlev and Gentofte, Herlev 2730, Denmark; ^f^Department of Medicine, Copenhagen University Hospital—Herlev and Gentofte, Herlev 2730, Denmark; ^g^Department of Clinical Medicine, Faculty of Health and Medical Sciences, University of Copenhagen, Copenhagen 2200, Denmark

**Keywords:** circular DNA, chromosomal amplification, oncogene, chromatin biology, colorectal cancer

## Abstract

Extrachromosomal circular DNA (eccDNA) are molecules that originate from chromosomal DNA but exist independently. While large eccDNA (ecDNA) contributes to tumorigenesis, the role of smaller eccDNA (<100,000 base pairs) in cancer remains unclear. Our analysis of 25 colorectal cancer (CRC) tumors and normal adjacent tissues revealed that eccDNA is significantly more abundant in tumor tissues, correlating strongly with chromosomal amplifications. The presence of whole intact genes on 1.29% of eccDNA was nonrandom. We identified 84 genes that recurred across tumors of multiple patients when present on eccDNA, with 19% of genes being cancer-associated. eccDNA-borne genes were often accompanied by increased expression, and their contribution to expression was much larger than that from linear amplifications and the larger ecDNA. The cytokine gene *CXCL5* exemplified this phenomenon, showing substantial copy-number increase and upregulation when present on eccDNA. Functional validation in cell lines showed that *CXCL5* eccDNA enhanced transcriptional output and immune cell recruitment function. The recurrence and overexpression of CRC-related genes on eccDNA indicate their selection in tumors, suggest that eccDNA can serve as an additional mechanism for dynamically influencing gene expression and is capable of conferring cancer phenotypes on cells. Analysis of chromatin landscapes revealed that eccDNA preferentially forms at sites of open chromatin and active transcription, with architectural boundaries marked by CTCF protein. Clinically, higher eccDNA levels correlated with poorer relapse-free survival in a small patient cohort. These findings suggest that circular DNA elements across the entire size spectrum participate in cancer evolution and warrant further investigation in larger cohorts.

## Significance

Colorectal cancer remains a leading cause of death worldwide, with tumor evolution and treatment resistance posing major clinical challenges. While large circular DNA molecules are known cancer drivers, the role of smaller extrachromosomal circular DNA (eccDNA) has been unclear. This study demonstrates that small eccDNA elements are significantly enriched in colorectal tumors and actively drive cancer progression by amplifying oncogene expression. We provide a functional proof that eccDNA can confer cancer phenotypes to cells, showing enhanced immune cell recruitment through *CXCL5* eccDNA. Patients with higher eccDNA levels show a correlation with poorer relapse-free survival in a small cohort, suggesting that the relationship between eccDNA abundance and clinical outcomes merits further investigation.

Cancer is a complex disease defined by its remarkable variability across multiple dimensions including histological, clinical, cellular, molecular, and epigenetic (reviewed in refs. 1 and 2). Among the emerging mechanisms contributing to tumor heterogeneity and adaptation is extrachromosomal circular DNA (eccDNA) of chromosomal origin. These circular DNA molecules exist outside chromosomal DNA and range dramatically in size from less than 100 base pairs (bp) to several megabases (3–5). The eccDNA family includes both small circles (<100 kb) from across the genome and larger, complex structures known as extrachromosomal DNA (ecDNA >100 kb) that often carry amplified oncogenes (3, 5–7). The role of ecDNA in driving tumor evolution through rapid oncogene amplification is well established (5, 8–11). This amplification can drive tumor heterogeneity and evolution by providing a mechanism for rapid gene copy number increase outside of chromosomal constraints. Consequently, ecDNA presumably contributes to the dynamic adaptability of cancer cells, influencing their growth, metastatic potential, and response to treatment (3, 5, 6, 9, 10, 12–14).

However, the population of smaller and more diverse eccDNA circles under 100,000 bp is poorly understood due to technical constraints (15). These circles originate from across the genome, especially from gene-rich chromosomes and repetitive sequence regions, and are found in both healthy somatic tissues and tumor tissues (TT) (3, 4, 11). Despite limited research, evidence suggests eccDNA may amplify oncogene copy numbers, generate novel oncogene isoforms, and serve as regulatory platforms for genetic adaptation (13, 15). The clinical relevance of eccDNA may be particularly apparent in colorectal cancer (CRC), one of the most prevalent malignancies in developed countries. Despite early detection through screening programs, CRC maintains high mortality rates and represents a significant health burden (16). Like many cancers, CRC exhibits substantial molecular and genetic heterogeneity (17–19). The disease progresses through dysregulation of key pathways: Wnt/β-catenin signaling drives early adenoma formation through APC mutations, RAS pathway mutations promote oncogenic survival signaling (reviewed in ref. 20) (21, 22), and PI3K/Akt/mTOR activation contributes to growth and treatment resistance (23). Additionally, chronic inflammation and immune evasion mechanisms facilitate CRC progression (24–26). For instance, CRC tumors often attract and activate immune cells, particularly neutrophils, through chemokines such as *CXCL5* (27–29). We chose CRC as a model to investigate eccDNA’s contribution to tumorigenesis, as CRC exhibits high levels of chromosomal instability and focal amplifications; offers availability of matched tumor-normal pairs from surgical resection; and has clinical relevance given CRC’s high mortality despite screening programs. The mechanisms underlying eccDNA formation and selection are likely not tissue-specific, making insights from CRC broadly applicable to understanding eccDNA biology across cancer types. The current understanding of eccDNA in CRC is mostly limited to primarily cell lines (30, 31), larger ecDNA (32), and small patient cohorts (33). Nevertheless, a recent study has shown a connection between eccDNA and CRC progression (34).

We hypothesize that eccDNA plays a critical role in CRC by facilitating increased expression of CRC-relevant genes with functional consequences for the tumors. We propose that such traits can lead to selection for CRC-relevant genes on eccDNA, leading to their overrepresentation. Finally, we suggest that chromosomal focal amplifications contribute to increased levels of eccDNA within tumors, with consequences for the patient. Using WGS and Circle-Seq of matched tumor and adjacent normal tissues from CRC patients, we demonstrate that tumors harbor significantly more eccDNA than normal tissue. Patients with the highest eccDNA levels experience poorer relapse-free survival, suggesting prognostic value for eccDNA abundance. Importantly, eccDNA carrying complete oncogenes correlates with increased gene transcription in TT, confirming functional impact. The recurrent observation of specific genes on eccDNA across multiple patients indicates consistent oncogene selection patterns. These findings establish eccDNA not merely as a reflection of genomic instability, but as an active driver of oncogenic processes in CRC progression and evolution. Similar patterns of eccDNA-mediated oncogene amplification and poor clinical outcomes have been documented across diverse cancer types, including neuroblastoma with MYCN-containing eccDNA, hepatocellular carcinoma with miRNA-17-92 amplicons, and medulloblastoma, suggesting that eccDNA represents a fundamental mechanism of cancer evolution that transcends tissue-specific boundaries and warrants systematic investigation in CRC (3, 35, 36).

## Results

### eccDNA Abundance Is Elevated in Colorectal TT Compared to Normal Adjacent Tissue.

**Fig. 1.**
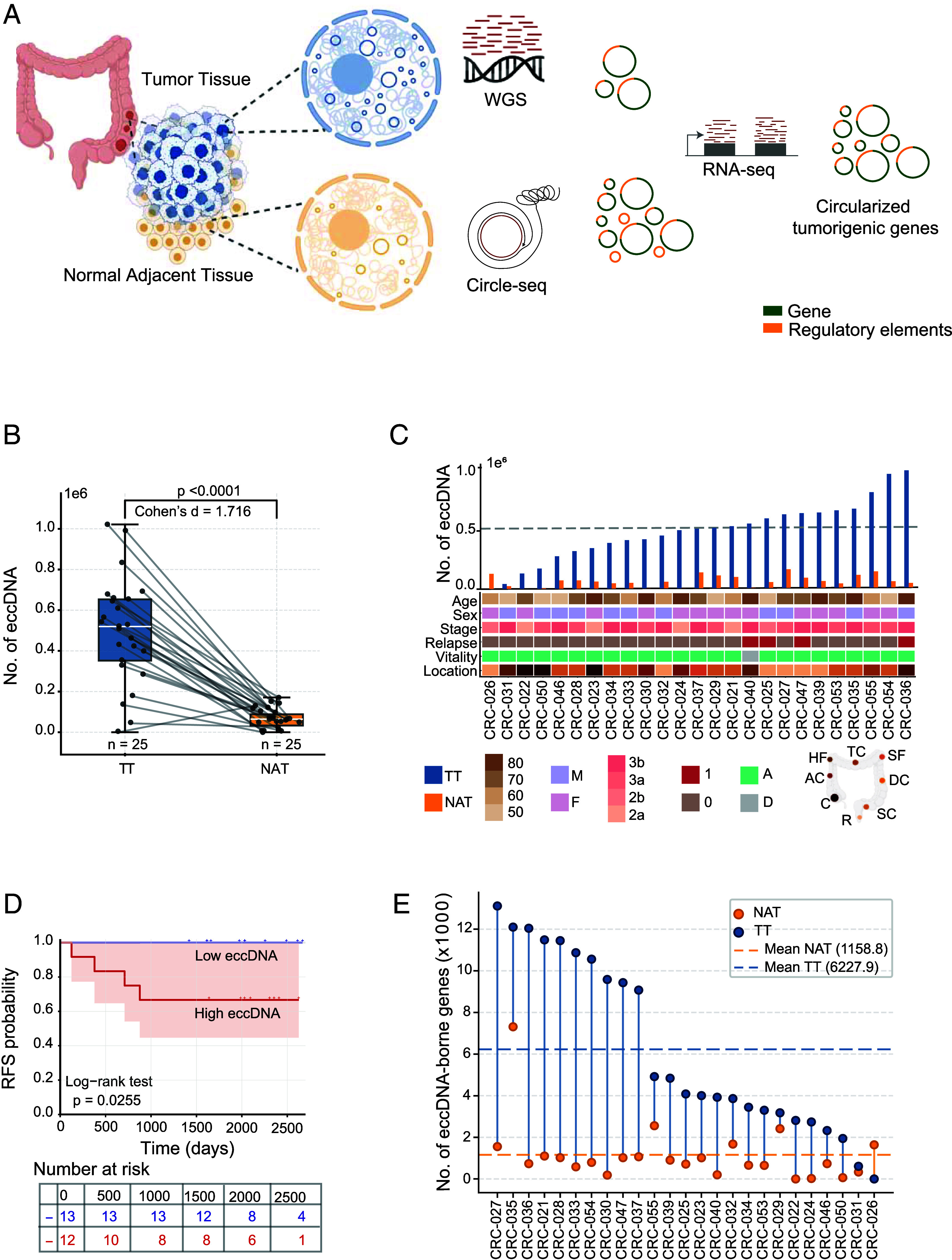
eccDNA in CRC. (*A*) Workflow integrating WGS, Circle-seq, and RNA-seq from matched tumor tissue (TT) and normal adjacent tissue (NAT) samples. (*B*) eccDNA counts in NAT (yellow, n = 25) vs. TT (blue, n = 25). TT: median = 523,684 (IQR: 385,467 to 654,628); NAT: median = 66,132 (IQR: 49,750 to 107,749); *P* = 2.58 × 10^−9^ (Wilcoxon rank-sum test); Cohen’s d = 1.716. Gray lines connect paired samples. (*C*) Patient-specific eccDNA profiles with clinical annotations (age, sex, stage, relapse, vitality, tumor location). (*D*) Kaplan–Meier curves for relapse-free survival stratified by median eccDNA count (log-rank *P* = 0.0255). (*E*) Paired comparison of eccDNA-harboring complete genes; dashed lines indicate means (TT: 6227.9; NAT: 1158.8).

To understand the landscape of circular DNA elements in CRC and investigate their effects on tumor phenotype, we performed circle-sequencing of eccDNA from TT and normal adjacent tissue (NAT) of 25 CRC patients removing both linear and mitochondrial DNA (37). RNA sequencing was performed on the matched TT and NAT samples to obtain corresponding transcriptome data. A subset of 12 TT samples was subjected to whole genome sequencing (WGS) to identify copy number variations (CNVs, Fig. 1*A*). *SI Appendix*, Table S1 provides the clinical characteristics of the sample cohort.

Circle-seq analysis identified 14,242,467 eccDNA elements across 25 samples, with 12,460,376 in TT and 1,782,091 in NAT samples. Notably, one tumor sample and one NAT sample had no detectable eccDNA. To address whether DNA quality, sequencing data quality, or sequencing depth was associated with the number of eccDNAs, we next performed correlation analyses between DNA quality metrics and eccDNA detection. For TT samples (n = 24), we found no significant correlation between DNA quality and eccDNA detection. DNA concentration showed no association with total eccDNA counts (Pearson r = −0.074, *P* = 0.73; Spearman ρ = 0.033, *P* = 0.88). Similarly, DNA yield showed no correlation with eccDNA counts (r = −0.074, *P* = 0.73). We observed significantly higher numbers of eccDNA in TT compared to NAT samples (TT: mean = 519,182, median = 523,684, IQR = 385,467 to 654,628; NAT: mean = 74,254, median = 66,132, IQR = 49,750 to 107,749; Wilcoxon rank-sum test, *P* = 2.45 × 10^−7^; Fig. 1*B*). This represents a sevenfold increase in eccDNA abundance in TT, with a large effect size (Cohen’s d = 1.716). Fig. 1*C* illustrates the eccDNA counts in paired tissues for each patient and their clinical characteristics. The eccDNA numbers did not correlate with the clinical characteristics of patients, such as age, sex, cancer stage, and tumor location (linear model, *P*-value = 0.7132). However, when we stratified patients into low (n = 13) and high (n = 12) eccDNA groups based on median tumor eccDNA count, patients in the low eccDNA group had no disease relapse (0/13, 0%) during the follow-up period, while 4 of 12 patients (33.3%) in the high eccDNA group experienced relapses. Fig. 1*D* illustrates the Kaplan–Meier curves of the two groups showing significant difference in relapse-free survival with log-rank *P* = 0.025. Due to the absence of events in the low eccDNA group, hazard ratio estimation was not feasible. The model showed good discriminative ability (C-index = 0.789, 95% CI: 0.687 to 0.891). Given the modest sample size and complete separation of events between groups, this survival analysis should be considered exploratory and requires validation in larger, independent cohorts.

### eccDNA Harbors Intact Genes.

We next aimed to determine if eccDNA serves a role beyond a structural genomic element by expressing intact genes within eccDNA (hereafter referred to as “eccDNA-borne genes”). We observed that the number of eccDNA-borne genes per TT sample ranged from 0 to 13,115, with a mean of 6,228 (median: 4,083; IQR: 3,178 to 10,559; n = 25; Fig. 1*E*). Notably, on average 1.29% harbored complete intact genes in tumor samples.

To assess whether complete genes were overrepresented on eccDNA, we compared the observed number of intact genes within eccDNA to expectations from 25 random genomic region datasets matched for size distribution and count per sample. This analysis revealed a burden-dependent pattern: Samples with lower total gene counts [log2 (counts + 1) < 3.5] showed enrichment of complete genes within eccDNA compared to random expectation, while samples with higher total gene counts [log2 (counts + 1) > 3.5] showed depletion, with random regions containing more genes than eccDNA (*SI Appendix*, Fig. S1*A*). Statistical analysis using permutation testing (n = 10,000) revealed that 20 of 24 samples showed significant deviation from random expectation (*P* < 0.05) (*SI Appendix*, Table S2).

To ascertain whether the increased eccDNA-borne genes could be attributed to higher gene density, we analyzed potential correlation between gene density and eccDNA-borne genes per chromosome. We observed no correlation (linear regression, R^2^= −0.240, −0.095 for TT and NAT, respectively) between gene density and eccDNA-borne genes (*SI Appendix*, Fig. S1*B*). These results suggest that eccDNA is a significant repository of genetic information and that factors other than gene density may contribute to the generation or retention of eccDNA within the genome.

To determine whether eccDNA-borne genes are shared between TT and NAT, we analyzed the overlap of eccDNA-borne genes in paired samples. The vast majority of tumor eccDNA elements were not detected in matched NAT. The median overlap was 4.5% (range: 0 to 22.3%), with 83.3% of patients (20/24) showing less than 10% overlap. One patient (CRCtx022) showed zero overlap between TT and NAT. This low overlap indicates that the eccDNA population in tumors is largely tumor-specific rather than reflecting shared tissue characteristics or general genomic instability affecting both TT and NAT.

### Genes on Circular DNA Exhibit Higher Expression than Linear DNA Counterparts.

**Fig. 2.**
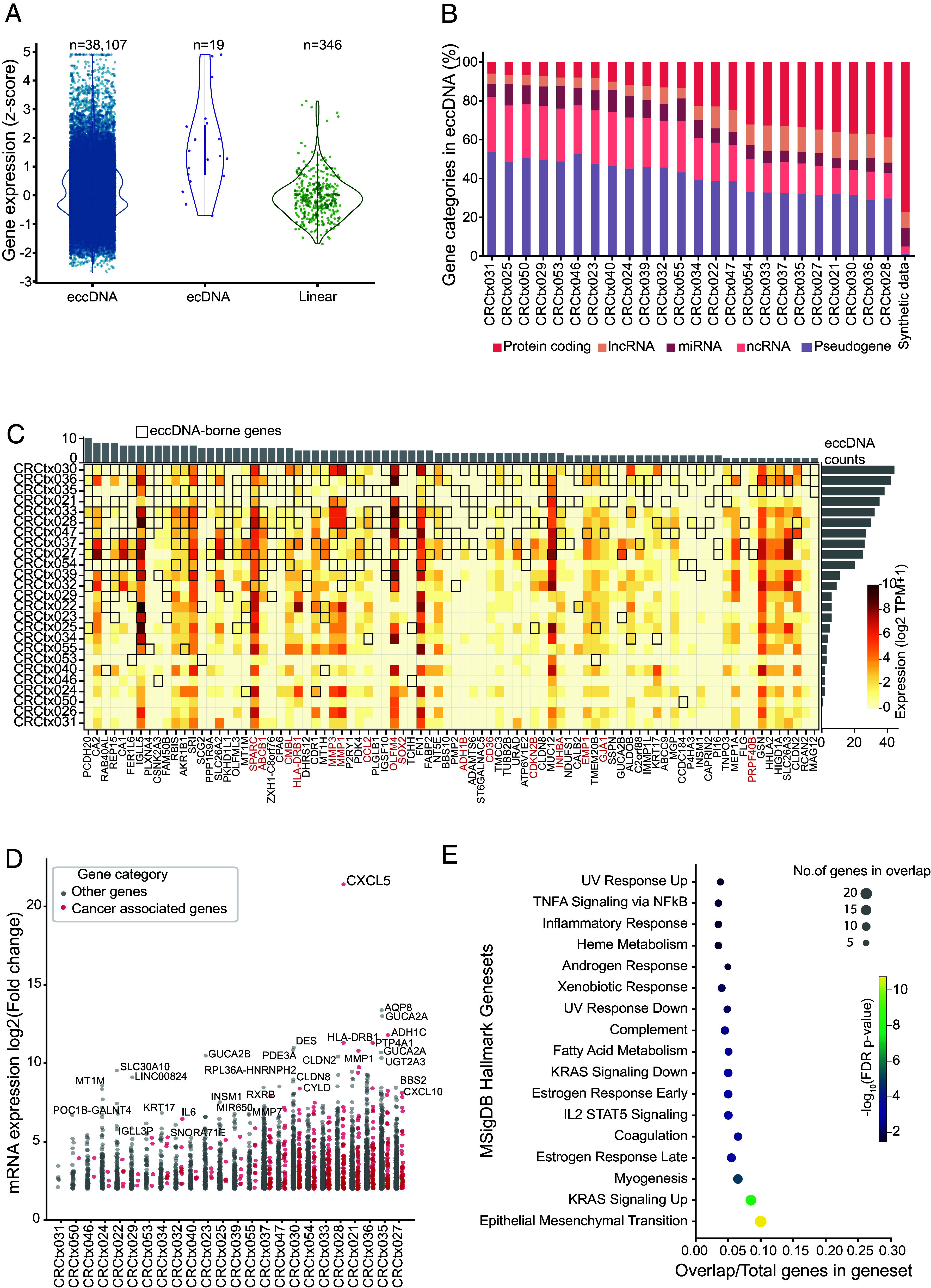
Characterization of eccDNA-containing genes. (*A*) Expression levels (z-score) of genes on eccDNA (n = 38,107), ecDNA (n = 19), and linear DNA (n = 346). (*B*) Gene categories of eccDNA-borne genes per sample. (*C*) Heatmap of eccDNA-borne gene frequency and expression (log2 TPM); black boxes indicate eccDNA presence; red labels denote cancer-associated genes. (*D*) Fold-change in eccDNA-borne gene expression (TT vs. NAT); red dots indicate cancer-associated genes. (*E*) MSigDB Hallmark enrichment analysis; circle size indicates overlap count; color indicates −log_10_(adjusted *P*-value).

Previous studies have shown that genes on circular DNA >100,000 bp (ecDNA) are often highly overexpressed in many tumors (3, 5, 7). To compare the contributions from eccDNA, ecDNA, and linear amplification to transcription, we employed Amplicon Architect to analyze the WGS data of 12 TT samples, identifying ecDNA and linearly amplified genes. This analysis identified 346 linearly amplified genes and 19 ecDNA-borne genes across the samples, in addition to 34,850 eccDNA-borne genes identified by the Circle-Seq analysis in the same samples. We then compared the expression levels of genes in linear DNA, ecDNA, and eccDNA using z-scores. Notably, while ecDNA-borne genes displayed the highest median expression levels, eccDNA contributed the most highly expressed circular genes overall (n = 38, 107 eccDNA genes vs. 19 ecDNA genes), with many eccDNA-borne genes reaching high z-scores in the upper tail of the distribution (Fig. 2*A*). Genes within eccDNA and ecDNA exhibited significantly higher expression levels than those in linear DNA (Kruskal–Wallis test *P* = 5 × 10^−07^). This finding underscores that circular DNA elements across the entire size spectrum enhance gene expression. We further examined the diversity and complexity of eccDNA-borne genes by analyzing their lengths and gene categories. Gene lengths ranged from 8 bp (*TRDD1*) to 2,473,620 bp (*RBFOX1*), with a mean size of 21,291 bp (*SI Appendix*, Fig. S1*C*). For protein-coding genes specifically, the size distribution (median: 25,554 bp; mean: 67,087 bp) closely resembled that of protein-coding genes across the genome (median: 27,411 bp; mean: 68,851 bp), suggesting no substantial size bias in eccDNA gene capture. In terms of gene categories, pseudogenes, protein-coding genes, snRNAs, and lncRNAs were the most frequently observed among eccDNA-borne genes (Fig. 2*B*).

### eccDNA-Borne Genes Are Associated with Carcinogenesis and Show Enhanced Expression.

Having established the high expression of eccDNA-borne genes, we investigated if certain genes on eccDNA had traits of selection by focusing on recurrent genes and their expression patterns in CRC. We first examined gene recurrence across our 25 tumor samples and identified 84 eccDNA-borne genes that appeared in ≥5 samples (~20% of samples) (Fig. 2*C*), among which, 19% (16/84) were cancer-associated, including well-characterized oncogenes such as *SPARC, ABCB1, CMBL, HLA-DRB1, MMP3, MMP1, CCL2, OLFM4,* and *SOX2*.

To assess functional relevance, we then examined expression changes in eccDNA-borne genes between paired TT and NAT samples. We identified 1,480 eccDNA-borne genes that were differentially expressed (|log_2_ fold change| > 1, *P* ≤ 0.05) between TT and NAT (Fig. 2*D*). Notably, 15.1% (223/1,480) were involved in known cancer-related functions, significantly more than the expected background proportion of 10.0% based on the total transcriptome (Binomial test, *P* = 6.4 × 10^−10^; *SI Appendix*, Table S3).

To further examine if eccDNA-borne genes were selected in the TT, we performed gene-set enrichment analysis on the top 500 upregulated eccDNA-borne genes using two gene sets from the MSigDB database: i) Hallmark and ii) C6 oncogenic gene sets. Hallmark gene set analysis revealed a significant enrichment of cancer-associated pathways (FDR-corrected *P* < 0.01), including epithelial–mesenchymal transition (EMT), Kirsten rat sarcoma viral oncogene homolog (*KRAS*) signaling, immune responses, and metabolism (Fig. 2*E*). The overlap between genes in each hallmark set and our upregulated gene list ranged from approximately 5 to 30%, with the EMT showing the highest overlap percentage. We found several significantly enriched C6 oncogenic gene sets as well, with a particular emphasis on 12 *KRAS*-related gene sets, along with gene sets associated with *TP53*, phosphatase and tensin homolog (*PTEN*), interleukin 2 (*IL2*), Wingless-related integration site (*WNT*) signaling, and activating transcription factor 2 (*ATF2*) pathways (*SI Appendix*, Fig. S1*D* and Table S4).

A striking example of eccDNA-driven transcriptional activation was observed in the *C-X-C motif chemokine ligand 5* (*CXCL5*) gene (Fig. 2*D*). Tumor sample CRCtx028 contained an eccDNA harboring *CXCL5* and gene expression showed a dramatic ~2^21^-fold increase compared to paired NAT. This exemplifies how even small eccDNA elements can drive profound transcriptional upregulation of cancer-relevant genes. Our analysis suggests that eccDNA-borne genes significantly influence gene expression in CRC, with recurrent oncogenes and differential expression patterns indicating a selective advantage in tumor progression. The enrichment of cancer-related pathways, particularly *KRAS* signaling and EMT, coupled with dramatic examples of gene upregulation like *CXCL5*, underscores the functional importance of eccDNA in tumor biology.

### Functional Validation Demonstrates eccDNA-Mediated Phenotypic Effects.

**Fig. 3.**
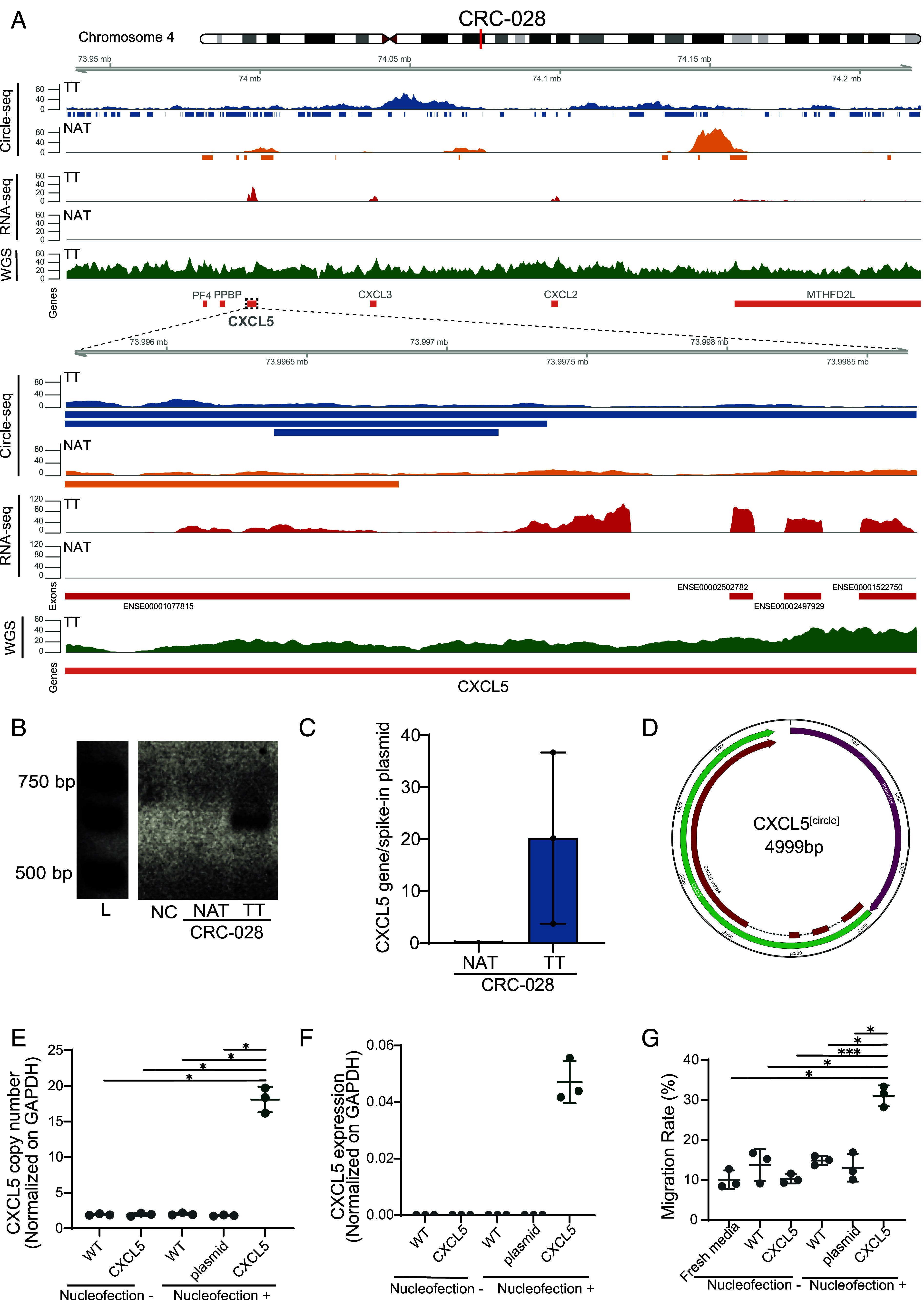
*CXCL5* circle expression. (*A*) Genomic profile of *CXCL5* locus (chromosome 4: 73.5 to 74.2 Mb) in patient CRC-028 showing Circle-seq, RNA-seq, and WGS coverage for TT and NAT. *Bottom* panels show a zoomed view (73.996 to 73.998 Mb). (*B*) PCR validation of [*CXCL5^circle^*] junction sites. L, 1 kb ladder; NC, negative control. (*C*) *CXCL5* copy number on eccDNA by qPCR, normalized to p4339 plasmid. (*D*) Synthetic [*CXCL5^circle^*] construct map (4,999 bp). (*E*) *CXCL5* copy number after transfection in SW620 cells (ANOVA *P* = 6 × 10^−10^). (*F*) *CXCL5* RNA expression posttransfection by droplet digital PCR. (*G*) THP1 migration assay using conditioned medium from [*CXCL5^circle^*]-transfected SW620 cells (ANOVA *P* = 5 × 10^−6^). For (*E* and *G*), *, *** indicate Tukey HSD family-wise error rate <0.05 and <0.001, respectively.

To investigate the potential direct association between eccDNA-borne genes and tumorigenesis, we focused on the *CXCL5* gene identified on eccDNA in the CRCtx028 CRC sample (Fig. 3*A*). We observed *CXCL5* to be expressed only in the TT sample, while no expression was recorded in the NAT sample. To confirm the presence and structure of the *CXCL5* eccDNA (hereafter referred to as [*CXCL5^circle^*]), we confirmed the junction site by PCR on agarose gel in the CRCtx028 sample for one possible circle (Fig. 3*B*). Quantitative PCR revealed that after removing linear DNA the copy number of the *CXCL5* gene was up to 20-fold higher in the tumor sample, while no *CXCL5* gene was found in the control NAT, (Fig. 3*C* and *SI Appendix*, Fig. S2 *A* and *B*). These results suggest amplification of *CXCL5* via eccDNA in the TT. To further understand the origin and mechanism of this overexpression, we attempted to identify allele-specific variants that could link the eccDNA to its chromosomal source through haplotyping. However, we did not observe any allele-specific markers on the eccDNA that would allow us to determine the haplotype from which it originated. This limitation prevents us from definitively connecting the observed *CXCL5* expression to a specific chromosomal allele.

Given these limitations in identifying the chromosomal origin of the natural *CXCL5* eccDNA, we decided to take a synthetic approach to directly test the functional implications of *CXCL5* eccDNA in CRC (*SI Appendix*, Fig. S2*C*). To achieve this, we developed a synthetic [*CXCL5^circle^*] (Fig. 3*D*) construct containing the 3,035 bp long *CXCL5* gene and its 1,845 bp promoter and transfected it into the colon cancer cell line SW620. Transfection of one million SW620 cells with 1.0 µg [*CXCL5^circle^*] led to an average copy number of 18.07 ± 1.45 *CXCL5*, while SW620 cells transfected with an eccDNA containing a random sequence carried only the expected two copies of *CXCL5* like the parental SW620 control (ANOVA F = 240.04, *P* = 6.92 × 10^−10^; Tukey’s HSD (honestly significant difference) for *CXCL5*_pos vs. plasmid_pos *P* < 0.001, and *CXCL5*_pos vs. Wild Type_negative WT_neg *P* < 0.001) (Fig. 3*E*). Cells exposed to the [*CXCL5^circle^*] without transfection did not increase the copy number of *CXCL5*, indicating that transfection successfully caused the [*CXCL5^circle^*] to enter SW620. We found that the transfected [*CXCL5^circle^*] was expressed in the SW620 cell line, while no expression of the *CXCL5* gene was detected in any of the controls (Fig. 3*F*).

The expression of *CXCL5* was also reflected at the phenotypic level. The *CXCL5* chemokine is known to induce migration of monocytes and other immune cells (38). We set up a Boyden Chamber cell migration assay in which THP1 monocytes were exposed to medium from SW620 with and without [*CXCL5^circle^*] and tested for their ability to migrate through a polycarbonate membrane. We found that spent medium from SW620 [*CXCL5^circle^*] cells induced a significantly higher migration rate of the monocyte cell line THP1 than spent medium from SW620 cells not transfected with [*CXCL5^circle^*] (Fig. 3*G*) (ANOVA F = 25.74, *P* = 5.04 × 10^−06^; Tukey’s HSD *CXCL5*_pos vs. *CXCL5*_neg *P* < 0.001, mean difference = 20.75). This enhanced migration was specific to the *CXCL5* eccDNA, as no significant differences were observed between control conditions including untransfected cells and cells transfected with control plasmid (Tukey’s HSD *P* > 0.05 for all control comparisons). In short, these experiments reveal that eccDNA in CRC is associated with increased expression of their constituent genes, and that expression of genes from synthetic eccDNA can allow cell lines to express cancer phenotypes. The ability of eccDNA to activate the *CXCL5* gene expression and influence immune cell recruitment further corroborates that eccDNA of all sizes can serve as a mechanism for dynamically controlling gene expression and that eccDNA has the potential to confer cancer phenotypes on cells.

### eccDNA Exhibits Size Periodicity, Chromosomal Bias, and Chromatin Boundary Associations.

We examined eccDNA size distribution across our samples, revealing distinct patterns between tumor and normal tissues. The median size of circles was 1,279 bp and 1,842 bp in TT and NAT, respectively. Furthermore, around 85% of the eccDNA detected was less than 2 kilobases (kb), and signatures of eccDNA were found in sizes up to 32 megabases (Mb) (*SI Appendix*, Fig. S3*A*).

**Fig. 4.**
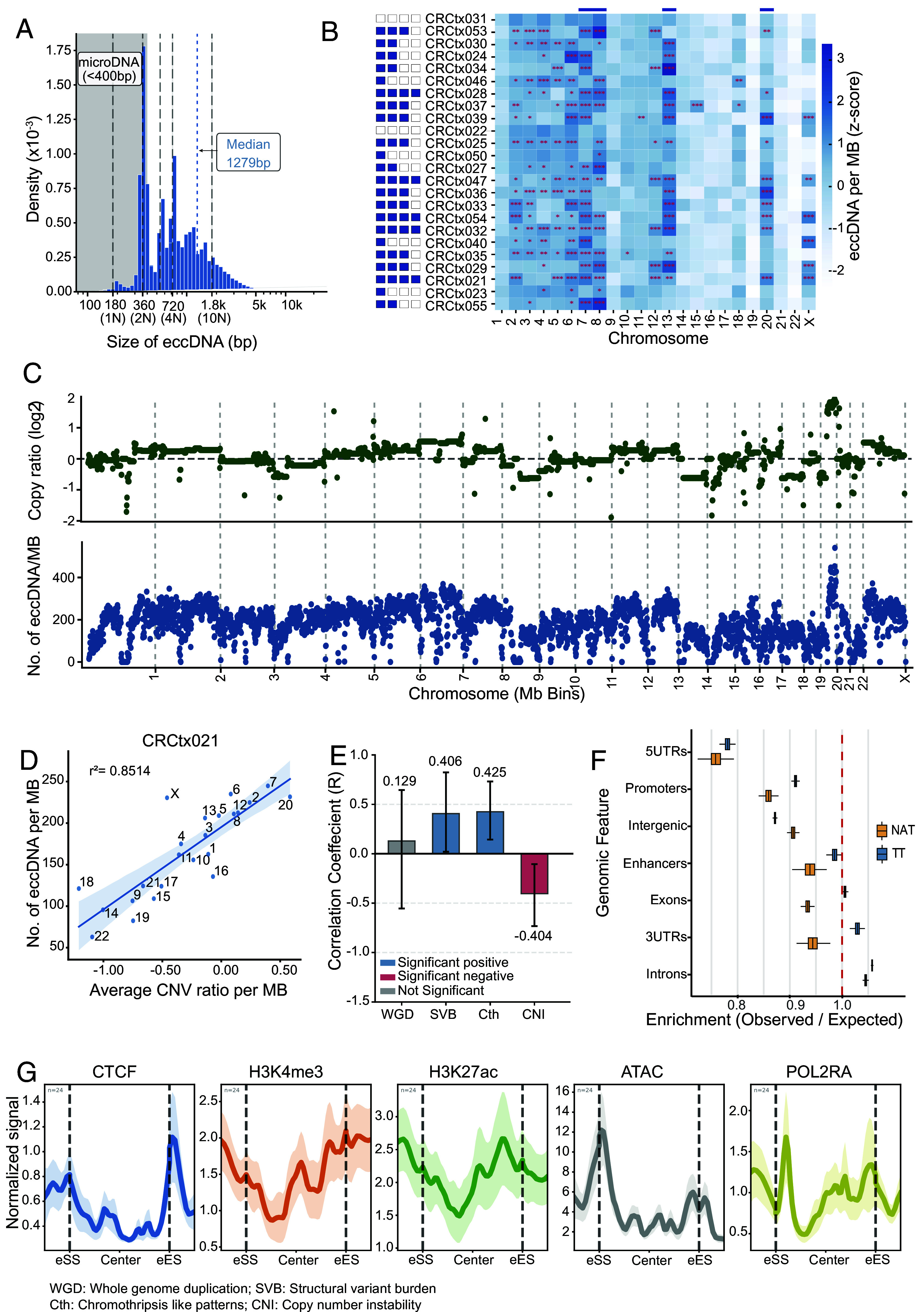
Genomic and epigenomic characteristics of eccDNA. (*A*) Size distribution of eccDNA relative to nucleosome units (1 N = 147 bp); median = 1,279 bp; the gray area indicates microDNA (<400 bp). (*B*) Chromosomal distribution of eccDNA (z-score per Mb). Asterisks denote significance (*P < 0.05, **P < 0.01, ***P < 0.001); blue boxes highlight enrichment in chromosomes 7, 8, 13, and 20. (*C*) Genome-wide eccDNA and copy number distribution for CRCtx021. (*D*) Correlation between eccDNA counts and copy number per chromosome in CRCtx021 (R^2^ = 0.8514). (*E*) Pearson correlations between eccDNA abundance and genomic instability features; error bars show 95% CI. whole genome duplication (WGD); SVB, structural variant burden; Cth, chromothripsis-like patterns; CNI, copy number instability. (*F*) Enrichment ratios of eccDNA across genomic elements for NAT (orange) and TT (blue); the dashed line indicates no enrichment (ratio = 1.0). (*G*) Chromatin feature signals across eccDNA regions; shaded areas represent 95% CI (n = 24 samples). eSS, eccDNA start site; eES, eccDNA end site.

The size distribution of eccDNA exhibited strong nucleosome periodicity, particularly in tumor samples (Fig. 4*A*). The most prominent peak was observed at 360 bp corresponding to the length of 2-nucleosome (2 N), containing 10.9% of tumor eccDNA compared to only 3.4% in NAT (3.2-fold enrichment, Chi-square test *P* < 0.0001). Secondary peaks at 3 N (540 bp) and 4 N (720 bp) positions showed 1.8-fold enrichments (both *P* < 0.0001). Overall, 40.2% of TT eccDNA aligned with nucleosome boundaries compared to 34.6% in NAT.

We examined the chromosomal distribution of eccDNA across the genome. Samples with no detectable eccDNA were excluded from this analysis. eccDNA mapped to all chromosomal regions, with notable enrichment in chromosomes 7, 8, 13, and 20 (Fig. 4*B* and *SI Appendix*, Fig. S3*B*). Positional enrichment analysis (per MB per chromosome) using chi-square goodness-of-fit testing revealed significant regional specificity in eccDNA formation, though enrichment patterns varied among samples. 10 samples (CRCtx021, 025, 027, 028, 029, 032, 035, 037, 047, 054) showed significant enrichment across all four chromosomes. Eight samples were enriched in three of the four chromosomes (CRCtx024, 030, 033, 034, 036, 039, 040, and 053) and four (CRCtx031, 046, 050, 055) in two. Only one sample (CRCtx023) showed enrichment restricted to chromosome 8, while another (CRCtx022) exhibited no significant overrepresentation in any of the four chromosomes, displaying a distribution consistent with random genomic origin.

In summary, 42% of samples exhibited enrichment in all four chromosomes, 33% in three, 17% in two, and 8% showed either single-chromosome or no significant enrichment.

We examined the relationship between eccDNA abundance and chromosomal copy number alterations using WGS data from 12 samples. Regression analysis between the number of eccDNA per chromosome per Mb and the average copy number per chromosome revealed a strong positive correlation (Fig. 4*C*). This relationship was particularly evident for chromosomes 7, 8, 13, and 20 - the same chromosomes identified in our regional bias analysis - which showed significant eccDNA enrichment across multiple samples.

In sample CRCtx021, elevated eccDNA per Mb was observed in chromosomes 7, 8, 13, and 20, directly aligning with corresponding copy number gains (R^2^ = 0.852) (Fig. 4*D*). This trend was maintained across all 12 samples [combined R^2^ = 0.714, *P* < 0.001, 95% CI: (0.672, 0.752); meta-analysis of 12 samples using Stouffer’s method, *SI Appendix*, Fig. S4*A*].

We analyzed three instability metrics across the 12 samples: WGD, structural variant burden, and chromothripsis-like patterns, to assess correlations with eccDNA abundance.

eccDNA abundance showed variable associations with global instability metrics (Fig. 4*E*). WGD events showed no correlation [R = 0.129, 95% CI: (−0.554, 0.645), *P* = 0.705]. Structural variant burden showed a moderate positive correlation [R = 0.406, 95% CI: (0.021, 0.824), *P* = 0.215], and chromothripsis-like patterns similarly correlated positively [R = 0.425, 95% CI: (0.144, 0.732), *P* = 0.192]. Copy number instability per chromosome showed a moderate negative correlation [R = −0.404, 95% CI: (−0.732, −0.105), *P* = 0.217].

We analyzed publicly available ATAC-seq (Assay for Transposase-Accessible Chromatin with sequencing) and ChIP-seq (chromatin immunoprecipitation with sequencing) data for six histone modifications and three transcription regulators from transverse colon samples (ENCODE *SI Appendix*, Table S5), using size-scaled boundary definitions (15% of eccDNA length, 200 bp to 1 kb range) and matched random controls (*SI Appendix*, Table S6).

Five chromatin features showed significant enrichment at eccDNA boundaries (false discovery rate FDR < 0.05). ATAC-seq demonstrated the strongest enrichment (1.057-fold, *P* = 2.27 × 10^−7^, Cohen’s d = 2.31). CCCTC-binding factor (CTCF) showed robust boundary enrichment (1.046-fold, *P* = 2.27 × 10^−7^, Cohen’s d = 2.09). Active transcription markers were also enriched: POLR2A (1.031-fold, *P* = 2.27 × 10^−7^, Cohen’s d = 3.60), H3K4me3 (1.021-fold, *P* = 2.27 × 10^−7^, Cohen’s d = 2.88), and H3K27ac (1.017-fold, *P* = 1.60 × 10^−3^, Cohen’s d = 0.72). Repressive marks (H3K9me3, H3K27me3), H3K36me3, and additional regulatory marks (H3K4me1, EP300) showed no significant enrichment.

Metaplots using 500 bp windows confirmed these patterns (Fig. 4*G*). Size stratification into small (1 to 5 kb), medium (5 to 20 kb), and large (>20 kb) eccDNA revealed distinct chromatin requirements (*SI Appendix*, Fig. S5 *A*–*D*). Small eccDNA showed dramatically stronger dependencies, with sixfold higher ATAC-seq signals and pronounced enrichment of all five significant marks. Medium and large eccDNA displayed progressively broader, less feature-specific patterns.

These analyses reveal that eccDNA exhibits nucleosome periodicity, preferential enrichment on chromosomes 7, 8, 13, and 20, and strong correlation with chromosomal amplifications. eccDNA boundaries are enriched for accessible chromatin, CTCF binding sites, and active transcriptional markers, with smaller circles showing stronger chromatin feature dependencies.

## Discussion

In this study, we demonstrate that CRC tumors carry large numbers of eccDNA with overexpressed recurrent oncogenes. We find that one such gene, *CXCL5*, confers tumor phenotypes when expressed from circular elements in a CRC cell line, and we show that in a small cohort, higher eccDNA levels in tumors correlated with disease relapse. This suggests that eccDNA can provide an evolutionary advantage to tumor cells by amplifying oncogenes or other critical genes. Many eccDNA-borne genes in the TT samples showed higher RNA expression levels compared to those in NAT samples, supporting the hypothesis that eccDNA can give rise to increased gene activity in cancer cells. It has been described before how oncogenes on the large ecDNA are correlated to higher RNA expression and tumor development (7). However, the evidence for the role of eccDNA <100,000 bp in tumorigenesis is limited and indirect. Henssen et al. have shown that genes on eccDNA are common and frequently overexpressed in neuroblastoma (3), and Ye and coworkers recently found a similar connection for eight liver tumors (35). To prove that the transcripts derived from the allele on eccDNA and not the chromosomal allele, Henssen and his team haplotyped transcripts from eccDNA-borne genes and found a strong bias toward the eccDNA-borne alleles. However, proof that expression from genes on eccDNA is responsible for tumor phenotypes has been lacking (10, 39). We reconstituted one of the eccDNA-borne genes with the highest increase in RNA expression level in a CRC cell line to test for this. One of the TT samples exhibited a significantly increased RNA expression level of the chemokine *CXCL5* and elevated gene copy numbers on eccDNA compared to the NAT. *CXCL5* is overexpressed in many tumors, including CRC, where it has been implicated in promoting tumor growth, metastasis, and angiogenesis (28, 29, 40). Cells with circular *CXCL5* DNA exhibited higher RNA expression levels and demonstrated the ability to affect neutrophils in a cell migration assay. Our findings support the hypothesis that gene amplifications on eccDNA can directly affect tumorigenesis by driving oncogene expression and altering tumor cell phenotypes. The *CXCL5* case illustrates that eccDNA-mediated gene amplification can occur independently of chromosomal copy number gains. While the chromosomal *CXCL5* locus remained diploid, qPCR on circular DNA fractions revealed substantial amplification on eccDNA. This demonstrates an alternative amplification mechanism that does not require prior linear DNA gains.

Despite these findings, the presence of genes on eccDNA does not universally correlate with elevated gene expression. A substantial proportion of highly expressed genes in our study were not located on eccDNA, and conversely, many genes found on eccDNA did not exhibit high expression levels. This implies that eccDNA is not the sole determinant of gene expression levels. Most eccDNA-borne genes did not show elevated expression in TT. This is in accordance with what may be expected if eccDNA follows Darwinian evolution, where the majority of eccDNAs have a neutral effect on cellular fitness, corresponding to limited functional consequence. A few have a positive effect on cellular fitness, favoring the cells carrying these particular eccDNAs. Furthermore, many circles may lack complete regulatory elements (promoters/enhancers) needed for expression. Additionally, eccDNA-driven expression might be restricted to specific cell types within heterogeneous tumors, while stochastic inheritance during cell division creates variable copy numbers that dilute bulk expression signals. However, when comparing RNA expression for genes found on eccDNA and ecDNA with the chromosomal linear amplifications, we observed significantly higher RNA expression for genes located on circle amplifications. This suggests that eccDNA provides a more effective platform for selective upregulation of certain genes compared to linear chromosomal amplifications. The effectiveness of circular elements to upregulate gene expression has been shown for the large ecDNA (3, 5, 7, 12), and the ability has been ascribed to missegregation of ecDNA in mitosis and altered chromatinization (7).

A hallmark of genomic instability in cancers, including CRC, is aneuploidy, which is frequently observed in solid tumors (reviewed in refs. 41–44). In our study, we found that the distribution of eccDNA origins in TT samples was skewed toward specific chromosomes, particularly chromosomes 7, 8, 13, and 20. Interestingly, our WGS analysis revealed amplifications and anomalies (partial aneuploidy) in these same chromosomes. Amplifications in chromosomes 7, 8, 13, and 20 are well established in CRC (45–50), while the loss or partial loss of chromosomes 8, 17, and 18 is also linked to CRC progression (47). Both focal amplifications and arm-level gains contribute to elevated eccDNA abundance, as seen on chromosome 12, where arm-level changes correlate with increased eccDNA signal. However, copy number changes alone cannot fully explain the observed patterns; the selective enrichment of protein-coding genes and specific chromatin features at eccDNA boundaries indicates that chromatin architecture and functional selection also shape the eccDNA population. Our study revealed that a substantial portion of eccDNA originates from enhancers and 5’ UTRs, while fewer fragments are derived from exons and introns. This contrasts with a previous CRC study by Chen et al. (33), which found a higher prevalence of eccDNA originating from exons and introns. Such variations across studies and cancer types reflect the diverse genomic landscapes from which eccDNA can emerge (33, 35, 51, 52). The differences in eccDNA origin may be influenced by the specific tissue types or the stage of cancer progression, highlighting the complexity of eccDNA biogenesis across cancers. The distinct enrichment of CTCF at eccDNA boundaries, along with the presence of open chromatin marks such as H3K4me3 and H3K27ac, suggests that chromatin organization may play a crucial role in the formation or stabilization of eccDNA. CTCF is well known for its role in establishing higher-order chromatin structure and regulating genomic architecture, often acting as a boundary element that demarcates chromatin domains (53–55). We hypothesize that CTCF binding sites may serve as preferred breakpoints during DNA damage events that generate eccDNA, similar to their role in translocation breakpoints (54). The enrichment of the active chromatin marks H3K4me3 and H3K27ac, which are typically associated with active enhancers and promoters, further supports the idea that eccDNA formation is biased toward regions of open and transcriptionally active chromatin (56, 57). These regions are often hotspots for regulatory activity (58, 59), suggesting that active chromatin features may predispose certain regions of the genome to eccDNA production (60, 61).

The observed eccDNA population appears distinct from apoptotic DNA fragments despite sharing nucleosome-periodic size distributions. Protein-coding genes are selectively enriched on tumor eccDNA (1.86-fold, *P* = 0.0059, Fisher’s exact test), whereas apoptotic fragmentation would produce proportional representation of all genomic elements. This selective retention of functional sequences suggests these circles are not merely byproducts of cell death. However, we cannot exclude that elevated eccDNA partly reflects increased cell death in tumors. Current methods do not permit single-cell resolution of the eccDNA origin to get a clearer picture.

Our data also revealed an increased amount of eccDNA in all tested CRC tumor samples compared to nontumorous samples, aligning with previous studies on colon and other tumors (33, 62, 63). The presence of eccDNA, particularly the ecDNA subpopulation, is linked to DNA damage, oncogene amplification, and cancer heterogeneity (5, 15, 64–67) and has also been documented in CRC (32, 33, 68). Furthermore, smaller eccDNA has been associated with DNA damage and genome instability (69). Notably, in our small cohort, we observed a correlation between higher eccDNA abundance and poorer relapse-free survival, though this finding requires validation in larger studies. In earlier works, cancer patients detected with the large ecDNA carrying oncogenes or having complex ecDNA had a lower 5-y survival in comparison with patients without the ecDNA (3, 12, 14, 70, 71). It has been shown that ecDNA serves as a fast adaptation tool for better survival for cancer cells, which leads to poorer survival for cancer patients (5). Our findings show that not only the big complex ecDNA molecules but also the broad-size (<100,000 bp) spectrum eccDNA are linked to patient survival and tumordevelopment. Importantly, the survival stratification reflects small eccDNA abundance rather than the presence of large ecDNA. With 12.4 million eccDNA elements detected across tumor samples and 85% smaller than 2 kb, the count metric is overwhelmingly dominated by small circles. Only two samples contained ecDNA, with discordant outcomes (one relapsed, one did not), indicating that large ecDNA presence does not explain the observed survival difference.

### Broader Biological Significance and Future directions.

Our findings demonstrate that eccDNA, including smaller circular DNA elements (<100,000 bp), plays a more significant role in cancer biology than previously recognized. We show that eccDNA can serve as a platform for oncogene amplification and expression in CRC, with direct implications for tumor phenotypes, as demonstrated through our *CXCL5* functional studies. The preferential formation of eccDNA from active chromatin regions and its correlation with chromosomal CNV suggest a complex interplay between genomic instability, chromatin state, and eccDNA biogenesis. The preliminary correlation between eccDNA abundance and relapse in our small cohort suggests that the clinical significance of eccDNA warrants further investigation in adequately powered studies. Understanding the mechanisms of eccDNA formation and its contribution to tumor evolution could lead to therapeutic strategies aimed at preventing or reducing eccDNA-mediated gene amplification in cancer cells. Future studies should focus on developing methods to specifically target eccDNA-bearing cells or prevent eccDNA formation, potentially offering new approaches to combat cancer progression and drug resistance.

### Limitations and Methodological Considerations.

The cross-sectional design precludes causal inferences about amplification–eccDNA relationships. The small cohort size limits statistical power for clinical associations and generalizability across populations. For the survival analysis specifically, the small cohort size and low event count preclude robust hazard ratio estimation, and these findings should be considered hypothesis-generating. The survival trend requires validation in larger, multi-institutional studies. Future multi-institutional studies with standardized Circle-Seq protocols are needed to confirm the prognostic value of eccDNA abundance in CRC. Technical challenges in detecting small eccDNA may influence observations, though validation experiments support biological relevance. While our findings are consistent with chromatin-guided eccDNA formation, alternative models including amplification-driven eccDNA excision cannot be excluded based on available data.

In conclusion, eccDNA represents a prevalent and functionally significant feature of CRC with distinctive chromatin associations and gene amplification capabilities across all size ranges. These findings provide a foundation for future mechanistic studies and potential therapeutic strategies.

## Materials and Methods

### Sample Collection and Study Design.

We analyzed 25 paired colorectal TT and NAT samples from the REBECCA biobank in Denmark (Ethics Committee approval VEK j.nr. H-2-2013-078). All samples were obtained from surgical resection specimens prior to any systemic therapy. No patients received neoadjuvant chemotherapy or radiation. Adjuvant treatment was administered following surgery per institutional guidelines and did not influence the molecular analyses performed on resection specimens. Clinical characteristics are detailed in *SI Appendix*, Table S1.

### eccDNA Purification and Sequencing.

Circular DNA was isolated from tissue samples through sequential Exonuclease V treatment and CRISPR-Cas9-mediated mitochondrial DNA removal, followed by rolling-circle amplification. The 100 kb threshold distinguishing eccDNA from ecDNA represents a field convention based on detection methodology rather than a strict biological boundary. Libraries were sequenced on Novaseq 6000 (2 × 150 bp paired-end). WGS was performed on 12 TT samples, and RNA-seq on all paired samples. Detailed protocols are provided in *SI Appendix, Supplementary Methods*.

### Bioinformatic Analysis.

eccDNA was identified using a mapping-based approach requiring at least two supporting reads with chimeric alignments or discordant mappings spanning breakpoints. Copy number analysis used CNVkit v0.9.9, with ecDNA identified by Amplicon Architect v1.2. RNA expression was quantified using Kallisto v0.50.1 with differential expression analysis by DESeq2. Chromatin feature enrichment was assessed using ENCODE ATAC-seq and ChIP-seq data from transverse colon tissue. Detailed computational parameters are provided in *SI Appendix, Supplementary Methods*.

### Functional Validation.

Synthetic *CXCL5* eccDNA was transfected into SW620 colon cancer cells by nucleofection. Copy number and expression were quantified by droplet digital PCR. Functional effects were assessed using Boyden Chamber migration assays with THP-1 monocytes. Complete experimental protocols are in *SI Appendix, Supplementary Methods*.

### Statistical Analysis.

Given the non-normal distribution of eccDNA counts, we report both mean ± SD to capture total burden and median (IQR) to represent typical values. Statistical comparisons were performed using nonparametric tests (Wilcoxon rank-sum). Recurrence-free survival was chosen as the primary endpoint due to the higher number of relapse events (n = 4) compared to death events (n = 1) during follow-up. Event-free survival analysis would be identical to relapse-free survival in this cohort, as no nonrelapse events occurred, and overall survival analysis was not feasible with only one death event. Kaplan–Meier curves were compared using log-rank tests, with all analyses performed in Python 3 and R v4.1.2.

## Supplementary Material

Appendix 01 (PDF)

## Data Availability

The datasets supporting the conclusions of this article are available in the Sequence Read Archive (SRA) under BioProject accession number PRJNA1186580 (72). The codes used to generate the results are available at Github (73). All other data are included in the article and/or *SI Appendix*.
