# Supplementary material for: Functional role of small extrachromosomal circular DNA in colorectal cancer
Source: Proc Natl Acad Sci U S A. 2026 Apr 2;123(14):e2523047123. doi: 10.1073/pnas.2523047123 (PMC13056112; doi:10.1073/pnas.2523047123)
Supplement: Supplementary file 1 — Appendix 01 (PDF) [file pnas.2523047123.sapp.pdf]

Supporting Information for

Functional Role of Small Extrachromosomal Circular DNA in Colorectal Cancer

Judith Mary Hariprakash[1#], Egija Zole[1#], Weijia Feng[1], Dan Hao[1], Lasse Bøllehuus Hansen[1], Nirmalya Bandyopadhyay[2], Marghoob Mohiyuddin[2], Sihan Wu[3], Astrid Zedlitz Johansen[4], Julia Sidenius Johansen[5,6,7], Birgitte Regenberg[1*]

Birgitte Regenberg

Email: bregenberg@bio.ku.dk

This PDF file includes:

Supplementary methods
Figures S1 to S5
Tables S1 to S7

Supplementary methods

Sample collection and description.

Our study cohort comprises 25 samples of paired colorectal tumor tissue (TT) and adjacent normal adjacent tissue (NAT) samples (N =50). The average age of the cohort sample was 66 years; with 14 females and 11 males with varying tumor stages (2a, 2b, 3a, 3b). The characteristics of the cohort are summarized in **Supplementary Table S1**. All samples were obtained from surgical resection specimens prior to any systemic therapy. No patients received neoadjuvant chemotherapy or radiation. Adjuvant treatment was administered following surgery per institutional guidelines and did not influence the molecular analyses performed on resection specimens.

This study was performed in accordance with the declaration of Helsinki. Patient tissue samples were obtained from the REBECCA biobank in Denmark and all samples were stored at -80 °C. The REBECCA study protocol was approved by the Ethics Committee of the Capital Region of Denmark (VEK j.nr. H-2-2013-078) and the Danish Data Protection Agency (j. nr. 2007-58-0015, HEH-014-044, I-Suite nr. 02771). Patients were recruited following ethical guidelines, and informed consent was obtained before sample collection. To ensure privacy, all samples underwent anonymization procedures before experimental processing. Anonymization involves assigning unique identifiers to each sample that are unlinkable to personal information.

Cell culture

The human SW620 colon cancer cell line and THP-1 monocyte cell line (mycoplasma tested) were cultured in RPMI 1640 Medium (ThermoFisher, USA), enriched with 10% Fetal Bovine Serum (ThermoFisher, USA) and 1% penicillin-streptomycin (ThermoFisher, USA), and incubated in a humidified incubator at 37 °C with 5% CO_2. Cells were cultured to 70–90% confluence in 10 cm² Petri plates and passaged every 2-3 days to maintain this cell density.

Plasmids and linear DNA quality control

For the quality control testing, we spiked-in a control mixture consisting of plasmids ((50,000 copies of p4339 (5064 bp), 10,000 copies of pBR322 (4,361 bp) (NEB, USA)).

All plasmids were maintained in Escherichia coli and purified with a standard plasmid midi-prep kit (NucleoBond® Xtra Midi, MACHEREY-NAGEL, DE). For quality control assessment, we used the standard protocol as per the manufacturer of qPCR assay with SYBR Green master mix (2x) (Applied Biosystems, USA) with primers from **Supplementary Table S7** and the results are shown in **Supplementary Figure 5e**.

Total DNA extraction, mitochondrial DNA and linear DNA removal from tissue samples

DNA from all tissue samples (6 mg) were extracted with MagAttract HMW DNA Kit (Qiagen, DE) as described in the manufacturer`s protocol. Total DNA from all samples was subjected to treatment with a plasmid-safe Exonuclease V for 5 days to remove linear DNA. We also removed mtDNA by using CRISPR/Cas9 system as described in Feng et al. 2022 with small changes (Feng et al., 2022). In short, at first, for 3 days prior mtDNA removal, we digested linear DNA by Exonuclease V. 20 µl of 10X NEB buffer 4, 20 µl of 10 mM ATP, 6 µl of Exonuclease V and 4 µl water (NEB, USA) were added per reaction and incubated for 3 days at 37 °C (every day adding extra 1.4 µl of 10X NEB buffer 4, 10 µl of 10 mM ATP and 3 µl of Exonuclease V), at the end heat-inactivated for 30 min at 70 °C). DNA was then cleaned using AMPure XP magnetic beads (Beckman Coulter, USA). mtDNA was linearized by Cas9 Nuclease, *S. pyogenes* kit (NEB, USA) according to the manufacturer's protocol. All gRNAs were designed as in Feng et al. 2022 (Feng et al., 2022). For CRISPR/Cas9 combining, 100 nM of Cas9 protein and 100 nM of sgRNA were mixed in a 1×NEB 3.1 buffer and incubated at 25 °C for 30 min. Then, sample DNA was added, mixed thoroughly, and incubated at 37 °C for 4 hours. Then, we inactivated the Cas9: sgRNA cleavage system by heating the sample for 10 min at 65 °C. After this step, Exonuclease V digestion was performed again to remove the now linearized mtDNA (2.1 µl of 10X NEB buffer 4, 15 µl of 10 mM ATP and 4.5 µl of Exonuclease V were added every day to the same reaction and incubated for 2 days at 37 °C, then heat-inactivated for 30 min at 70 °C). Followed by DNA cleaning using 1.8x ratio of AMPure XP magnetic beads that were added to each sample, mixed by pipetting 10x and incubated at room temperature for 5 min. The samples were put on a magnetic rack for 3 min, following which the supernatant was discarded. The samples were then washed twice with 200 µL 75-80% ethanol and dried until the beads were slightly moist. The beads were then resuspended in

15 µL of 10 mM Tris-HCl (pH=8) (ThermoFisher, USA) buffer and mixed by pipetting 10x, before being incubated for 5 min at 50 °C. This was followed by a 2 min bead separation step on the magnetic rack, after which 13 µL of the eluate was taken out and transferred to a clean new 1.5 mL DNA Lobind tube (Eppendorf, DE) (2 µL were left behind). The elution step was then repeated using 12 µL elution buffer and combined with the other elute for a final sample volume of 25 µL. For DNA concentration measurements and quality control, 10 µL of each sample was transferred to a new PCR tube.

Rolling-circle amplification of eccDNA for Circle-Seq for tissue samples

All the purified eccDNA samples (15 µL) were then used as a template for Φ29 polymerase reactions (4BB™ TruePrime® RCA Kit) (4basebio PLC, UK), which was added in accordance with the manufacturer's instructions and incubated for 48 h at 30 °C.

RNA extraction from tissue samples

RNA was extracted from the last 25 paired samples. For tissue under 5 mg, we used the miRNeasy Tissue/Cells Advanced Micro Kit (Qiagen, DE), and for tissue up to 30 mg, we used the miRNeasy Tissue/Cells Advanced Mini Kit (Qiagen, DE), as stated in the manufacturer`s protocol. For quality control, we used the Agilent RNA 6000 Pico Kit (Agilent Technologies, USA).

eccDNA, whole genome DNA, and RNA library preparation and sequencing

For the eccDNA sequencing, a portion of each Φ29-amplified DNA sample was diluted to 15 ng/µL in 10 mM Tris-HCl (pH=8) for a total volume of 100 µL and then sonicated (4 cycles of 20sec/30sec (on/off time) using a Bioruptor (Pico, Diagenode, USA).

For the WGS, total DNA was diluted to 15 ng/µL in 10 mM Tris-HCl (pH=8) for a total volume of 100 µL and then sonicated in the same manner as for plasma samples. The libraries were then prepared using the NEBNext Ultra II DNA Library Prep Kit for Illumina and NEBNext Multiplex Oligos for Illumina (NEB, USA) in accordance with the manufacturer's protocol. For the sonication step and library quality control, we used the Agilent DNA 1000 Kit (Agilent Technologies, USA).

Following library preparation, all eccDNA samples were multiplexed and sequenced on a Novaseq 6000, S4 flow-cell (Illumina, USA) as 2×150-nucleotide paired-end reads, with

an average of 157 million single reads per sample. WGS samples were sequenced with Novaseq 6000, S1 flow-cell as 2×150-nucleotide paired-end reads, with an average of 62 million single reads per sample (all sequencing was done at Rigshospitalet, Denmark).

For RNA-seq, we used NEBNext rRNA Depletion Kit v2 (Human/Mouse/Rat) and NEBNext Ultra II Directional RNA Library Prep Kit for Illumina; for barcoding, we used NEBNext Multiplex Oligos for Illumina (96 Unique Dual Index Primer Pairs) (NEB, USA) as stated in manufacturer`s protocol. RNA was multiplexed and sequenced on a Novaseq 6000, SP flow cell as 2×75-nucleotide paired-end reads, with an average of 25 million single reads per sample (all sequencing was done at Rigshospitalet, Denmark).

eccDNA copy number variation detection by qPCR

To check for copy number variations for CXCL5 gene with high RNA expression in cancer tissue samples, we used qPCR assay to measure relative copy number in TT and NAT samples by using SYBR™ Green PCR Master Mix (2x) (Thermo Scientific, USA) and a primer pair from **Supplementary Table S6**. For qPCR, after a denaturation step at 95 °C for 10 min, samples were incubated for 40 cycles at 95 °C for 15 s and at 60 °C for 30 s. For eccDNA-purified DNA concentration was unknown as it was too low to detect by Qubit. Serial dilution standard curves for qPCR were used to determine CNV. Relative CN in eccDNA-purified DNA=CN CXCL5 gene/CN of spike-in plasmid. Each sample was run in triplicates. A melting curve analysis was performed to verify the specificity and identity of the qPCR products. Removal of linear DNA was confirmed during the same qPCR runs by ALB gene primers and with an additional PCR reaction with Cox5B primers using DreamTaq PCR Master Mix (2X) (Thermo Scientific, USA) standard protocol with modifications by adding 5% of DMSO and 2 nM of $MgCl_2$, and 40 cycles and visualized on 2% agarose gel (SeaKem LE Agarose, Lonza Group, CH) with 60x GelRed Nucleic Acid Gel Stain (Biotium, USA) (**Supplementary Figure 9 c,e**).

[CXCL5^{circle}] eccDNA validation with PCR

To confirm the presence of the [CXCL5^{circle}] in the CRCtx028 sample, we performed PCR targeting the junction site of the circle. The PCR was conducted using DBdirect™ PCR Gel Mix SuperSens (2x) (DIANA Biotechnologies, CZ) according to the manufacturer's instructions, with [CXCL5^{circle}] primers (final concentration of 5 nM) listed in

Supplementary Table S6. We used rolling-circle amplified DNA from the CRC-028 patient`s tumor tissue sample and normal adjacent tissue sample, diluted to a concentration of 45 ng/µL (2.25 ng/µL final concentration). The PCR amplicons were visualized on 1% agarose gel (SeaKem LE Agarose, Lonza Group, CH) with 60x GelRed Nucleic Acid Gel Stain (Biotium, USA).

Reverse transcription

iScript™ cDNA Synthesis Kit (Bio-Rad, USA) was used for the reverse transcription according to the manufacturer's guidelines. Briefly, 20 µl reaction system was prepared, including 500 ng of total RNA, 4 µl of 5x iScript reaction mix, 1 µl of iScript reverse transcriptase and nuclease-free water. To initiate the reverse transcription operations, the temperature was first set to 25 °C for 5 min, then raised to 46 °C for 20 min, and finally increased to 95 °C for 1 min to inactivate the reaction.

Droplet Digital PCR

Droplet Digital PCR (ddPCR) reactions were set up using the subsequent methodology in a 20 µl reaction system, including 5 ng of genome DNA, 2 pmol of each prime, 10 µl QX200TM ddPCR™ EvaGreen Supermix (Bio-Rad, USA), and sterile water. The PCR mixture was then put in a Bio-Rad QX200 Droplet Generator (Bio-Rad, USA) to produce droplets. Subsequently, a 40 µl droplet was placed onto a Bio-Rad 96-well PCR plate and sealed with foil, heating it to 170 °C for 4 seconds. Next, 98 °C for 5 min, 40 cycles of 95 °C for 30 s and 60 °C for 1 min, followed by 4 °C for 5 min, 90 °C for 5 min, and a 4 °C hold, were used for the PCR. For every phase, a ramp rate of 2 °C/s was employed. The droplets were then read using the Bio-Rad QX200 ddPCR apparatus. The data visualization and analysis software utilized was Bio-Rad QuantaSoftTM Analysis (Version 1.0.596).

Nucleofection

CXCL5 circular DNA was synthesized by GenScript Europe company (Rijswijk, NL), and delivered to SW620 cells using the SE Cell Line 4D-Nucleofector™ X Kit S (Lonza Group, CH) in accordance with the manufacturer's instructions. Briefly, the SW620 cells were trypsinized, quenched with culture medium (1% penicillin-streptomycin and 10% FBS), and 10^6 cells were counted and centrifuged at 500 rpm for 5 min. After a single PBS buffer

wash, the cells were resuspended in 20 μl of the electroporation solution, and 1 μg of CXCL5 circular DNA was added. Then, the cell suspension was moved to a nucleocuvette and electroporated in a Lonza 4D-Nucleofector device under the CM130 program. Subsequently, the cells were seeded in a six-well plate with pre-warmed culture media and underwent two days of culturing.

Boyden Chamber cell migration assay

Two days post-nucleofection, the culture medium of the SW620 cells was transferred to the bottom chamber of a 24 mm Transwell® with an 8.0 μm pore polycarbonate membrane insert (Corning, USA). Simultaneously, $2x10^6$ THP-1 cells were seeded in the upper chamber of the same Transwell® for a 6-hour migration. After the migration period, the upper chambers were removed, and the cell numbers in the lower chambers were counted.

RNA expression analysis

The quality of the RNA-sequencing data was assessed using Fastqc (RRID: SCR_014583) v0.11.9 (https://www.bioinformatics.babraham.ac.uk/projects/fastqc/), following which the low-quality reads and adapters were trimmed using fastp (RRID: SCR_016962) v0.20.1 (https://github.com/OpenGene/fastp). The quantification of the transcripts was obtained using Kallisto (RRID: SCR_016582) v0.50.1 (https://pachterlab.github.io/kallisto/) as Transcripts per Kilobase Million (TPM) based on the GRCh38 reference genome. DEseq2 was used to calculate the fold change for each tumor tissue sample in comparison to the collectively grouped normal adjacent tissue samples.

Gene set enrichment analysis

500 differentially expressed genes (DEGs) from DEseq2 were used for the gene set enrichment analysis (GSEA). Two different datasets of the MSigDB resources (http://www.gsea-msigdb.org/gsea/msigdb/index.jsp) Hallmark and C6 (oncogenic gene sets) were used through the GSEA (RRID: SCR_003199). Gene sets with an FDR-corrected $p < 0.01$ were considered significantly enriched, and the results were plotted using custom Python scripts.

eccDNA identification

We employed a mapping-based approach to identify chromosomal-derived eccDNAs, following the pipeline described by Feng et al. (2022). An event was considered a circle if supported by at least two sequence reads indicating the presence of a chimeric alignment, discordantly paired-end mappings, or soft-clipped reads spanning the circle breakpoint. Notably, recurrent eccDNAs such as mitochondrial DNA were counted as single events. The identified eccDNAs were assigned further confidence levels based on coverage criteria: PASS1: At least 95% of positions within the putative eccDNA circle were covered by reads. PASS2: Mean coverage within the circle was at least twice the mean coverage in neighboring regions in addition to fulfilling the PASS1 criteria. The 100 kb threshold distinguishing eccDNA from ecDNA represents a field convention based on detection methodology rather than a strict biological boundary. Given the non-normal distribution of eccDNA counts, we report both mean ± SD to capture total burden and median [IQR] to represent typical values. Statistical comparisons were performed using non-parametric tests.

Gene annotations were based on the GRCh38 assembly, obtained from ENSEMBL (http://ftp.ensembl.org/pub/release-110/gtf/homo_sapiens/Homo_sapiens.GRCh38.110.gtf.gz). We used BedTools v2.30.0 (RRID: SCR_006646) intersect to determine gene containment within eccDNAs. An eccDNA was considered to contain a whole gene if the gene's start position was greater than the eccDNA's start position and the gene's end position was less than the eccDNA's end position.

WGS analysis

Raw sequencing reads were assessed for quality using FastQC (RRID: SCR_014583) v0.11.9. Adapter sequences and low-quality bases (Phred score < 30) were trimmed using Trimmomatic (RRID: SCR_011848) v0.38. Clean reads were aligned to the human reference genome (GRCh38) using BWA-MEM (RRID: SCR_010910) v2.2.1 with default parameters. The resulting SAM files were converted to BAM format, sorted, and indexed using SAMtools (RRID: SCR_002105) v1.18. PCR and optical duplicates were marked using Picard MarkDuplicates (RRID: SCR_006525) v2.9.1.

Copy Number Variation Analysis

Copy number analysis was performed using CNVkit (SCR_021917) v0.9.9. We used duplicate-removed BAM files as input, with the hg38 human reference genome as the baseline. To improve accuracy and reduce false positives, we applied an access list to exclude problematic regions of the genome based on a modified version of the ENCODE blacklist regions for hg38. Gene-level annotations were incorporated using RefSeq gene definitions (refFlat.txt) to provide biological context to the detected CNVs. Custom Python scripts were used for further downstream analysis and visualization.

Whole Genome Doubling Detection

WGD events were identified using a multi-criteria approach based on CNVkit-derived copy number profiles. For each sample, we calculated the fraction of the genome showing copy number gains (log2 ratio > 0.58, corresponding to copy number ≥ 1.5) by summing the length of gained segments divided by total analyzed genome length. WGD status was assigned when ≥50% of the genome showed coordinated copy number increases and ≥10 chromosomes displayed gains in >30% of their length. A quantitative WGD score was calculated as the genome-wide fraction of gained segments to enable correlation analysis with eccDNA abundance.

Structural Variant Burden Quantification

Structural variant burden was estimated from copy number breakpoint density using CNVkit segmentation data. Breakpoints were identified as positions where consecutive segments on the same chromosome showed copy number changes exceeding 0.3 in log2 space. For each sample, we calculated: (1) total estimated breakpoints across all chromosomes, (2) copy number variance as a measure of segmental instability, and (3) per-chromosome copy number instability defined as the variance of log2 ratios within each chromosome. A composite SV burden score was computed by combining breakpoint density and copy number variance (breakpoints + variance × 100) to provide a single metric for correlation analysis.

Chromothripsis-like Event Detection

Chromothripsis-like events were identified by analyzing patterns of clustered copy number oscillations within individual chromosomes. For each chromosome, we calculated the

frequency of high-amplitude copy number changes ($|\Delta log2| > 0.5$) between consecutive segments. Chromosomes were classified as chromothripsis-like when >30% of segments showed high-amplitude oscillations, indicating extensive local rearrangement. A chromothripsis score was calculated as the proportion of chromosomes per sample showing chromothripsis-like patterns, enabling quantitative correlation with eccDNA abundance.

Genomic Instability Profile Integration

To assess combined genomic instability effects, we generated a composite instability score by standardizing and summing WGD scores, SV burden scores (normalized by dividing by 100), and chromothripsis scores. This combined metric was correlated with total eccDNA abundance to test whether multiple instability mechanisms collectively influence eccDNA formation patterns.

Correlation confidence intervals were calculated using bootstrap resampling (10,000 iterations) to account for small sample size effects and provide robust estimates of statistical significance. Bootstrap confidence intervals were prioritized over Fisher-z transformation due to their superior performance with small samples. Correlations were considered statistically significant if the 95% bootstrap confidence interval excluded zero.

Amplicon Architect

Regions exhibiting copy numbers greater than five were selected as putative intervals for ecDNA reconstruction using Amplicon Architect (v1.2). Amplicon Architect was used to assemble focal amplifications, identifying and characterizing the structure of amplified genomic regions. The resulting assemblies were further classified using Amplicon Classifier (v0.4.11) (Luebeck et al., 2023), which was run with default parameters to categorize the amplicon structures.

eccDNA Chromosomal Distribution

To determine the genomic distribution of eccDNA across chromosomes, we first normalized the number of eccDNA occurrences per chromosome to per Mb, followed by z-score normalization to account for differences in chromosome length. To assess whether the occurrence of eccDNA in each chromosome was significantly higher than expected by

chance, a chi-square goodness-of-fit test was performed, comparing observed eccDNA distributions to expected distributions based on chromosome length.

Copy Number Variation Analysis

CNV data were processed from CNVKit results in BED file format. CNV per Mb was calculated by binning the hg38 reference genome using the bedtools makewindows function, followed by intersecting the log2 ratio values from the CNV BED files with genomic bins. This approach provided normalized CNV measurements across all chromosomes for correlation analysis with eccDNA density.

Correlation and Regression Analysis

Linear regression analysis was performed to quantify relationships between eccDNA density per chromosome and corresponding genomic metrics (copy number alterations and gene density) using the linregress function from scipy.stats. Pearson correlation coefficients were calculated to assess the strength of linear relationships, with R^2 values determined to quantify the proportion of variance explained by each model.

Meta-Analysis

For multi-sample analysis, individual correlation results were combined using Stouffer's method for p-value combination and weighted averaging for R^2 values. Individual p-values were converted to Z-scores and combined using sample size weights ($w_i = n_i - 2$, representing degrees of freedom for linear regression): $Z_{combined} = (\Sigma w_i \times Z_i) / \sqrt{(\Sigma w_i)}$. The combined R^2 was calculated as a weighted average: $R^2_{combined} = (\Sigma w_i \times R^2_i) / (\Sigma w_i)$. Confidence intervals for combined effect sizes were calculated using Fisher's Z transformation with appropriate back-transformation to the correlation scale. For meta-analysis results, combined p-values were calculated using Stouffer's method, and 95% confidence intervals were reported for all combined effect sizes.

Genomic Instability Metrics

Genomic instability was quantified using four metrics: (1) whole genome duplication fraction (proportion of genome with copy number gains), (2) structural variant burden (breakpoint density and altered genome fraction), (3) chromothripsis-like patterns

(oscillating copy number changes), and (4) copy number instability (variance of log2 ratios per chromosome).

Pearson correlations between eccDNA abundance and instability metrics were calculated for 11 samples with complete data. Given the small sample size, 95% confidence intervals were estimated using bootstrap resampling (10,000 iterations) rather than parametric methods. Correlations were considered significant if bootstrap confidence intervals excluded zero. Bootstrap methods provide robust significance testing for small-sample correlations without distributional assumptions.

Chromatin mark enrichment

Publicly available ATAC-seq and ChIP-seq data generated from human transverse colon tissue were downloaded from ENCODE as BigWig and Bed files. Accession numbers for the datasets used are provided in **Supplementary Table S5**

eccDNA Filtering and Size Stratification

eccDNA regions were filtered to include only those ≥1kb in length to ensure reliable boundary analysis. For size stratification analysis, eccDNAs were classified into three categories: small (1-5kb), medium (5-20kb), and large (>20kb). This stratification was performed to investigate potential size-dependent chromatin formation mechanisms.

Size-Scaled Boundary Definitions

To address the biological heterogeneity of eccDNA sizes, we implemented adaptive boundary definitions rather than fixed-size regions. Boundary regions were defined as 15% of each eccDNA's length, with minimum and maximum limits of 200bp and 1kb, respectively. This approach ensures biologically relevant boundary analysis across the diverse eccDNA size spectrum while maintaining statistical power. Internal regions were defined as the middle 50% of each eccDNA, excluding boundary regions to prevent overlap.

Matched Random Controls

Size-matched random genomic controls were generated to validate the specificity of chromatin enrichments. For each eccDNA in each sample, we generated three random genomic intervals of identical length, ensuring a minimum 5kb distance from any eccDNA

to avoid confounding effects. Random intervals were distributed genome-wide proportional to chromosome sizes, creating a robust null distribution for comparison.

Signal Quantification and Normalization

Chromatin signals were extracted from BigWig files using pyBigWig, with mean signal values calculated for each defined region (boundary, internal, external). To account for local chromatin context differences, signals were normalized relative to ±10kb flanking windows around each eccDNA. Fold enrichment was calculated as the ratio of boundary signal to internal signal for each eccDNA.

Statistical Analysis

Boundary enrichment was assessed using the Wilcoxon signed-rank test, comparing fold enrichment values against the null hypothesis of no enrichment (fold change = 1). Effect sizes were calculated using Cohen's d approximation. To control for multiple testing across ten chromatin features, we applied the Benjamini-Hochberg false discovery rate (FDR) correction with $\alpha = 0.05$. Statistical analyses were performed in Python using scipy.stats.

Metaplot Generation

To visualize spatial chromatin patterns, we generated metaplots showing normalized signal intensity across eccDNA regions. Each eccDNA was divided into 100 bins with additional 20-bin flanking regions (500bp upstream/downstream). Profiles were aggregated across all samples and smoothed using Gaussian filtering ($\sigma = 1.0$) for visualization. Size-stratified metaplots were generated separately for each eccDNA size category.

Genomic annotation of eccDNA regions

To assess whether eccDNA formation occurs preferentially in specific genomic features, we performed matched sampling analysis comparing observed eccDNAs with size- and chromosome-matched random genomic regions. We randomly sampled 10% of eccDNAs from each sample (or 50,000 regions for samples with >500,000 eccDNAs) and generated an equal number of random regions matching the exact chromosome and size distribution of the sampled eccDNAs. This process was repeated for 1,000 iterations to generate robust statistics. Genomic annotations for hg38 were obtained using the annotatr package v3.19 (10.18129/B9.bioc.annotatr), including promoters (1-5kb upstream of transcription start

sites), 5' UTRs, 3' UTRs, exons, introns, intergenic regions, and FANTOM5 permissive enhancers. For each iteration, we calculated the number of eccDNAs and matched random regions overlapping each genomic feature. Enrichment was calculated as the ratio of observed to expected overlap frequencies, normalized by feature size (regions per Mb). Statistical significance was assessed using the empirical distribution of enrichment values across iterations, with p-values calculated as the proportion of iterations where enrichment deviated from 1.0. Multiple testing correction was performed using the Benjamini-Hochberg method. To examine size-dependent patterns, we stratified eccDNAs into small (<1 kb) and large (>10 kb) categories and repeated the analysis. All analyses were performed separately for tumor and normal samples to identify tissue-specific patterns. Confidence intervals (95%) were calculated from the distribution of enrichment values across iterations.

Clinical features and survival analysis

Clinical characteristics of patients, including age, sex, cancer stage, and tumor location, were compared to the number of eccDNAs present in tumor tissues using a linear regression model. Patients were further stratified into eccDNA-high and eccDNA-low, based on a cutoff of 5×10^6 eccDNAs, representing the average eccDNA number across the cohort. For survival analysis, patients were stratified into low and high eccDNA groups based on the median tumor eccDNA count. Recurrence-free survival was defined as the time from surgery to first documented disease recurrence or last follow-up for patients without recurrence. Survival curves were constructed using the Kaplan-Meier method and compared between groups using the log-rank test. Cox proportional hazards regression was initially attempted to calculate hazard ratios; however, due to complete separation in the data (zero events in the low eccDNA group), hazard ratio estimation was not feasible. Model discrimination was evaluated using Harrell's concordance index (C-index) with 95% confidence intervals calculated using bootstrap methods. Recurrence-free survival was chosen as the primary endpoint due to the higher number of relapse events (n=4) compared to death events (n=1) during follow-up. Event-free survival analysis would be identical to RFS in this cohort, as no non-relapse events occurred, and overall survival analysis was not feasible with only one death event. R packages survival (v.3.4.0) and survminer (v.0.4.9) were used for the analysis.

Random Dataset Generation and Permutation Analysis

To generate random datasets for comparative analysis with the eccDNA regions, we utilized the read_regions and randomize_regions functions. Using the randomize_regions function, we generated 25 random datasets for each sample with random genomic regions, allowing for overlaps and maintaining the per-chromosome distribution of the original eccDNA regions. The number of eccDNA-borne genes was computed for each random dataset using bedtools intersect. The difference in means between the observed tumor data and the randomly generated datasets was employed as the test statistic for evaluating enrichment.

The NumPy.random.shuffle in Python function was utilized to shuffle the combined dataset (which included both the tumor and random data) and calculate the difference in means for the permuted groups. This shuffling process helps assess how the observed differences could arise under the null hypothesis that no true effect exists. We executed 10,000 permutations to generate a distribution of differences in means, which serves as a reference to evaluate the observed test statistics. The p-value was calculated by determining the proportion of permuted differences as extreme as or more extreme than the observed difference in means. A lower p-value indicates stronger evidence against the null hypothesis, suggesting significant enrichment of eccDNA-borne genes in the tumor samples compared to the random datasets.

Gene Expression Analysis

We identified genes within eccDNA from Circle-seq data and linearly amplified genes from Amplicon Architect analysis of WGS data across 12 tumor samples. Genes were classified into three groups: eccDNA-borne, linearly amplified, and non-amplified (control). RNA expression data were Z-score normalized for all identified genes across corresponding samples. To evaluate differences in gene expression between groups, a Kruskal-Wallis test was performed using the scipy.stats module.

Functional Annotation of Differentially Expressed Genes

To determine the functional relevance of the differentially expressed genes, we analyzed the subset of DEGs for their involvement in cancer-related functions. A comprehensive list

of 2,769 cancer-related genes was obtained from four online sources: Network of Cancer Genes and Healthy Drivers (http://ncg.kcl.ac.uk/), Cancer Genetic Web (https://www.cancer-genetics.org/), Oncogene database (https://ongene.bioinfo-minzhao.org/browse_gene.html), and Federal Office for Consumer Protection and Food Safety https://zag.bvl.bund.de/onkogene/index.jsf?dswid=8028&dsrid=703. A binomial test was employed using scipy.stats.binomtest function from Python to evaluate whether the number of cancer-related DEGs within the eccDNA dataset was significantly higher than expected by chance.

Statistical analysis

All statistical analyses were conducted using Python 3 or R version 4.1.2. Continuous variables were compared between tumor and normal tissues using the Wilcoxon rank-sum test due to non-normal distribution of eccDNA counts. Effect sizes were calculated using Cohen's d. Correlations between eccDNA levels and clinical characteristics were assessed using linear regression models using the following formula:

```
m2 <- lm(efile$ecDNA_TT ~ efile$Age + as.factor(efile$Sex) + as.factor(efile$Stage) + as.factor(efile$Tumor.location), data = efile)
```

Statistical Significance

Statistical significance was set at $p < 0.05$ for all analyses. All tests were two-sided unless otherwise specified. Results are presented as mean ± standard deviation or median (interquartile range) as appropriate. The statistical significance of differences between [CXCL5^{circle}] experimental groups was assessed by one-way ANOVA and subsequent Tukey's HSD (honestly significant difference) test using the python scipy library.

Figures

Supplementary figure 1

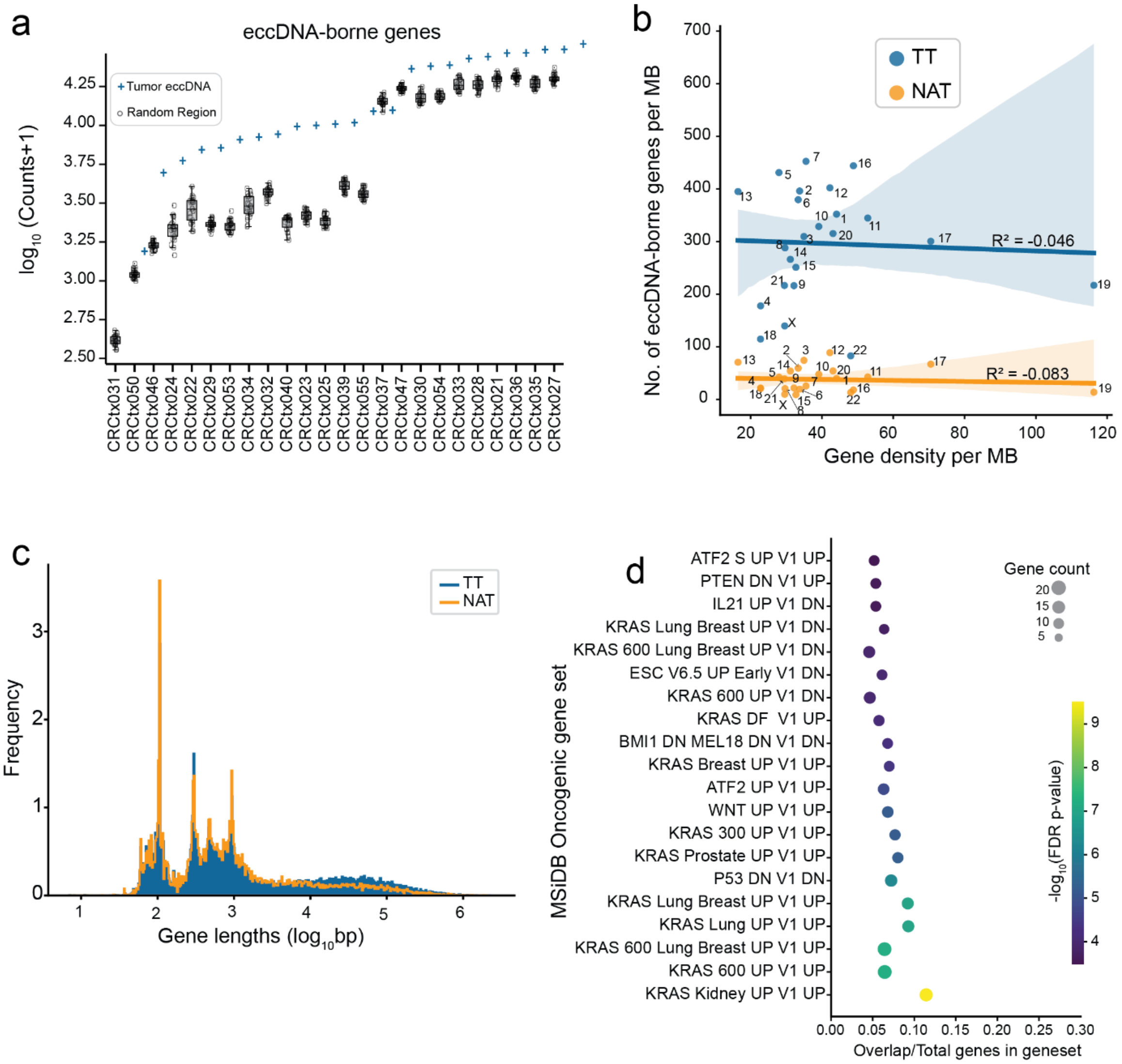

Supplementary Figure 1. Characterization of eccDNA-borne genes. (**a**) Comparison of eccDNA-borne genes and random genomic regions across patient samples. The y-axis shows $\log_{10}$(counts+1) of eccDNA, while the x-axis represents different TT samples. Blue plus sign indicates the number of eccDNA-borne genes in TT sample. The box plot shows the distribution of the number of eccDNA-borne genes in 25 synthetic datasets for each sample. Each box represents the interquartile range (IQR) containing the middle 50% of the data, with the horizontal line inside the box indicating the median. The whiskers extend to the minimum and maximum values within 1.5 times the IQR. Individual data points are

plotted as dots, representing specific samples. **(b)** Regression plot showing the no. of eccDNA-borne genes per MB to the gene density per MB. Each point represents an individual chromosome. Blue and yellow dots represent TT and NAT samples respectively. The blue and yellow line indicates the linear regression fit, with the shaded area showing the 95% confidence interval. Coefficient of determination (r^2) for each category is mentioned in the figure. **(c)** Distribution of gene lengths (log_{10} base pairs) for eccDNA-borne genes in TT (blue) and NAT (yellow) samples. The x-axis represents gene length, and the y-axis shows frequency. (**d**) The scatter plot shows the over-representation of differentially expressed eccDNA-borne genes in the MSigDB Oncogenic gene set. The x-axis represents the ratio of the overlapping genes to the total number of genes in the pathway. The size of the circle denotes the number of genes in overlap, and the color shows the negative logarithmic adjusted p.

Supplementary Figure 2

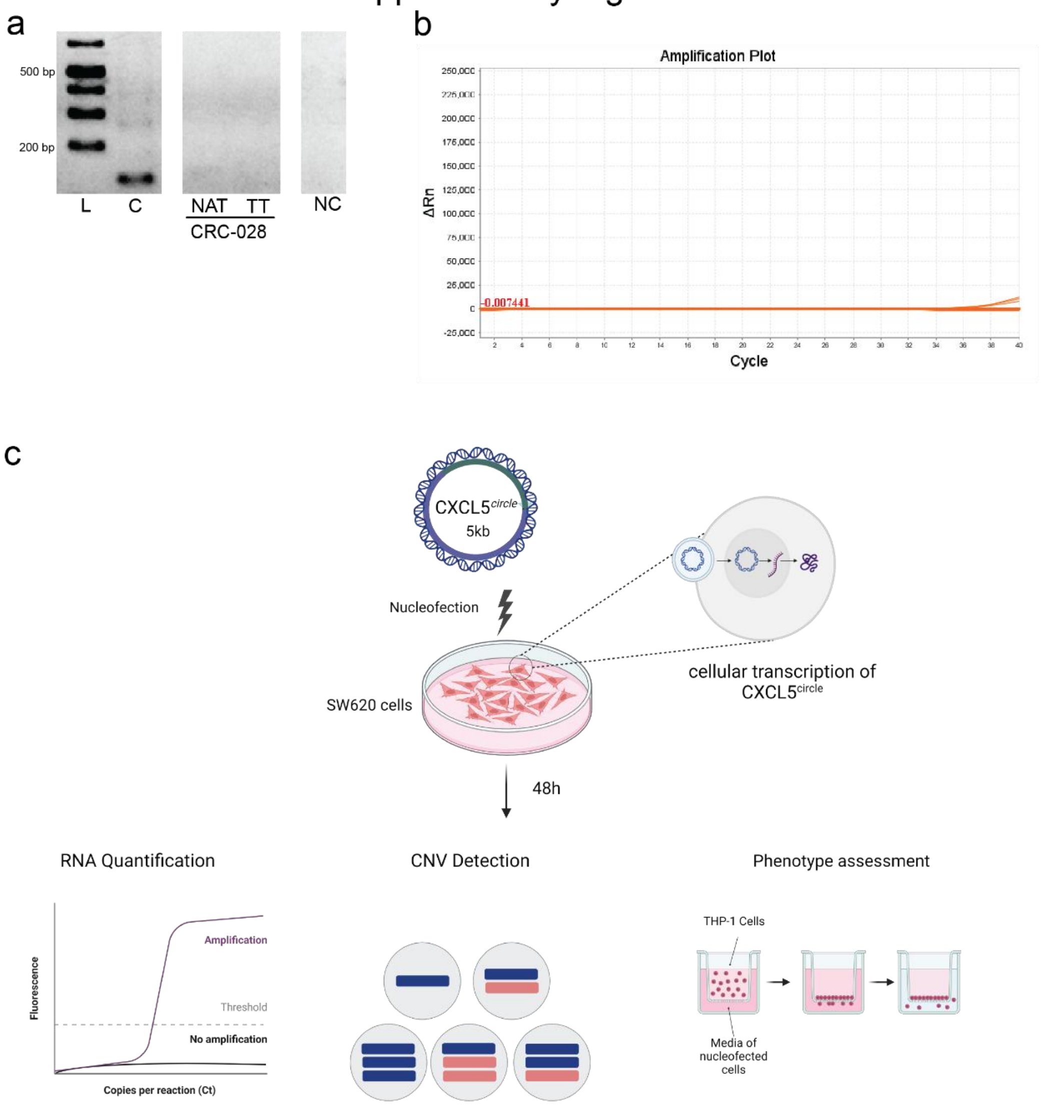

Supplementary Figure 2. Experimental validation. (a) An agarose gel electrophoresis of PCR amplification products was performed to verify the presence of linear DNA following the linear DNA removal process. L, 1 kb plus ladder; TT, tumor samples; NAT, non-tumorous samples; PC, positive control; NC, negative control; bp, base pairs. **(b)** qPCR amplification of the presence of linear DNA following the linear DNA removal process. The ΔRn value represents the difference between the Rn value of the experimental reaction and the Rn value of the baseline signal produced by the instrument. Rn, normalized reporter signal. **(c)** Experimental workflow for studying the effects of [CXCL5^{circle}] in SW620 cells: Nucleofection: The top of the figure shows a representation of the CXCL5

synthetic construct with 3,035 bp long gene with its 1,845 bp promoter being introduced into SW620 cells via nucleofection. Incubation: The cells are then incubated for 48 hours. Analysis: The workflow branches into three types of analyses: RNA Quantification, CNV Detection and Phenotype assessment using Boyden Chamber cell migration assay in which monocytes (THP1 cells) exposed to medium from SW620 with and without [CXCL5^{circle}] to test their ability to migrate through a polycarbonate membrane.

Supplementary Figure 3

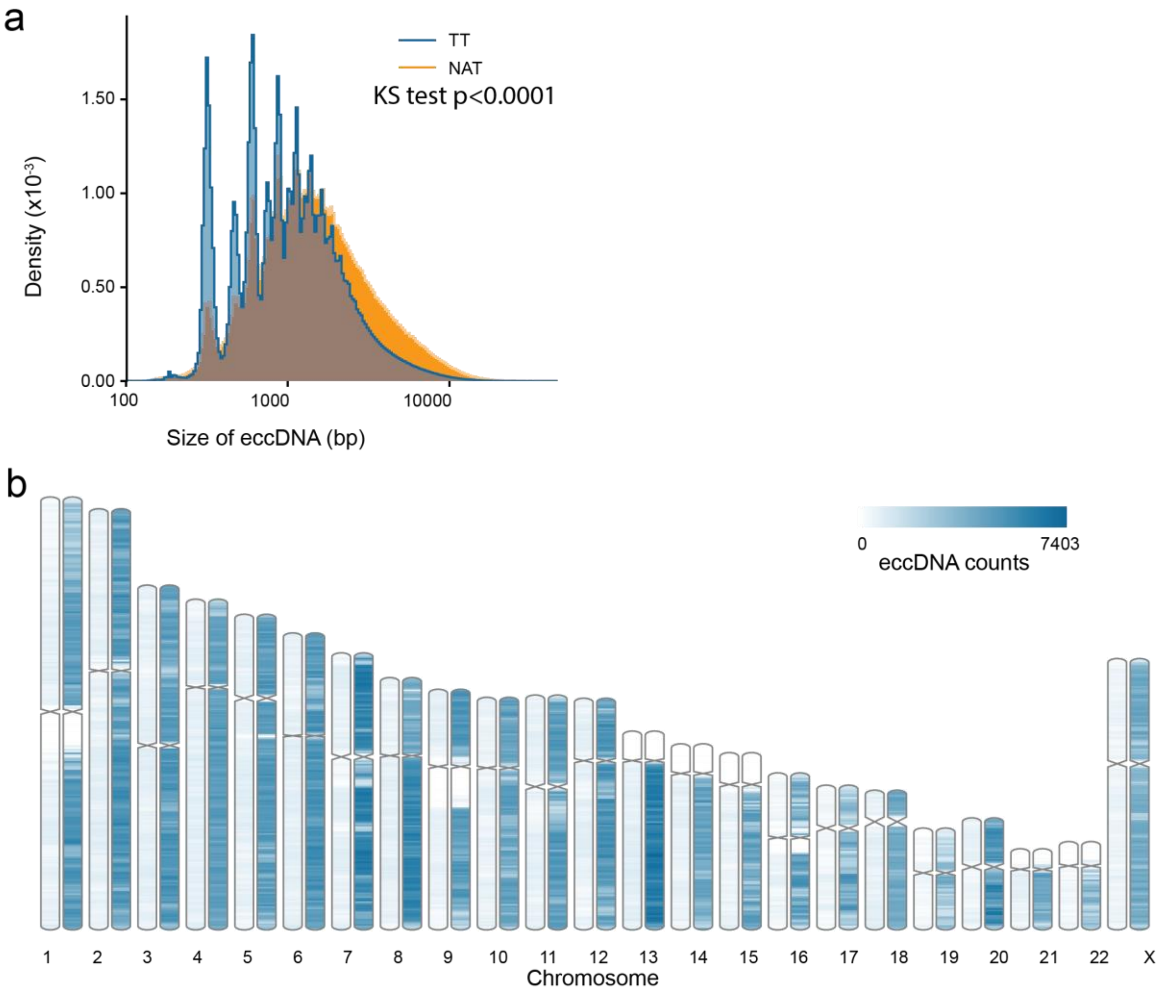

Supplementary Figure 3. Characterization of eccDNA Profiles (a) Density plot of eccDNA size distribution in TT and NAT samples. **(b)** Distribution of combined eccDNA counts across chromosomes in 25 NAT and TT samples. The bar plot displays eccDNA counts for each chromosome (1-22 and X) in NAT (left bar) and TT (right bar) samples. The x-axis represents chromosomes, while the y-axis shows eccDNA counts. The color intensity corresponds to eccDNA abundance, with darker blue indicating higher counts, as shown in the legend (0 to 7403 eccDNA counts.

Supplementary Figure 4

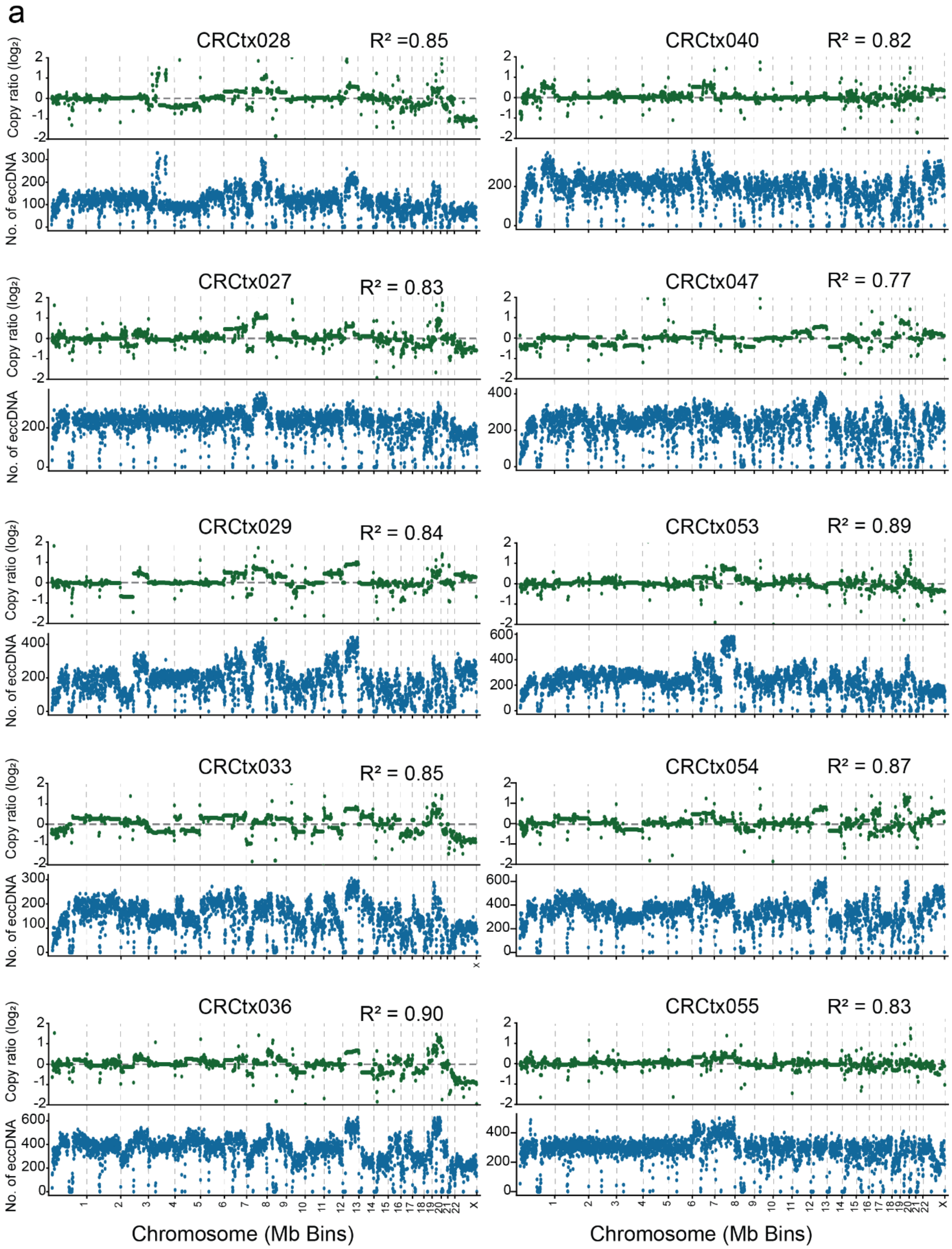

Supplementary Figure 4. Comparison of eccDNA and copy number (a) Genome wide distribution of eccDNA count per MB to the copy number ratio. The top panel (green)

shows the $\log_2$ copy number ratio across chromosomes per MB obtained from CNVKit, while the bottom panel (blue) represents the corresponding eccDNA counts per MB for the samples in the cohort. The coefficient of determination (r^2) determined by regression analysis is labeled in the figure.

Supplementary Figure 5

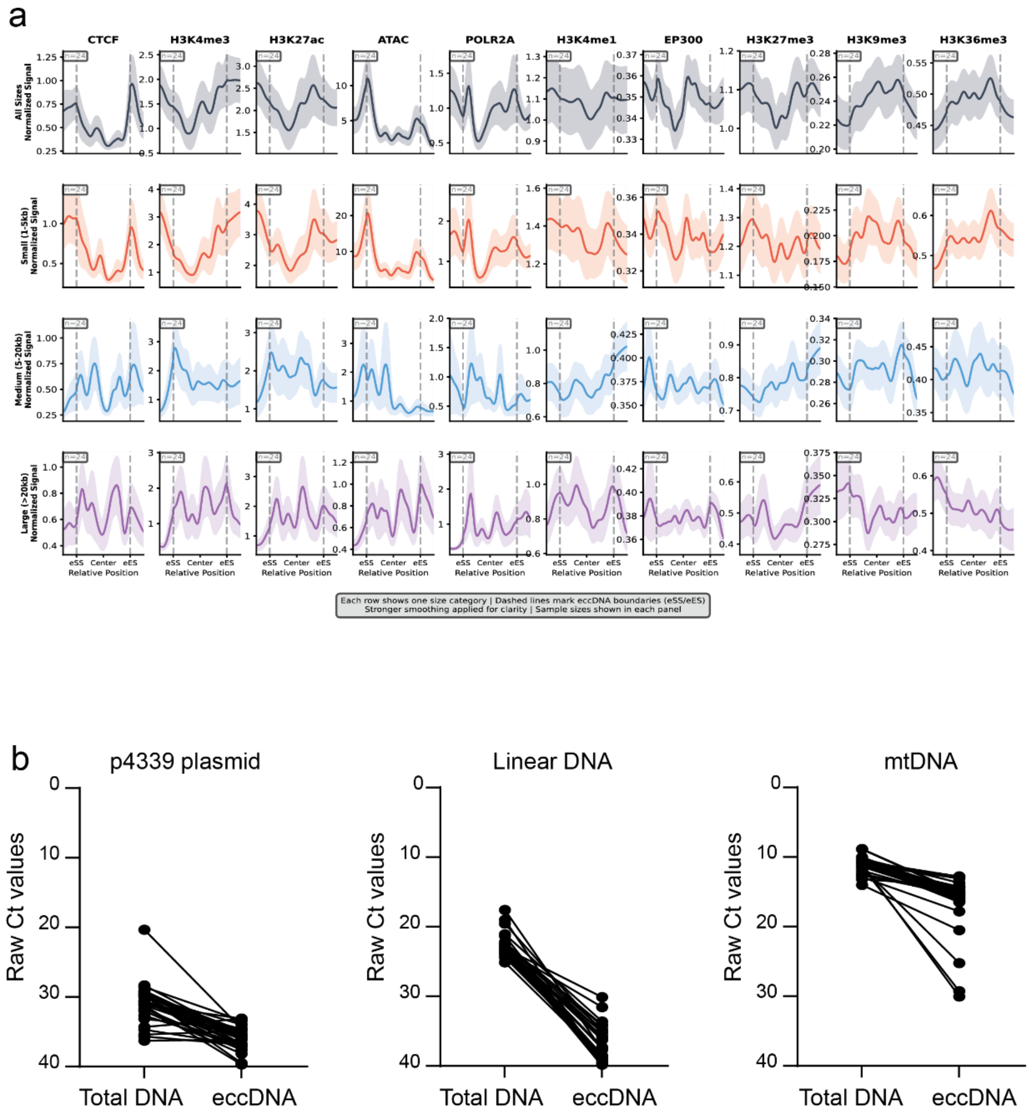

Supplementary Figure 5. Tracks show normalized read density for: CTCF, H3K4me3, H3K27ac, ATAC, POL2A, H3K4me1, EP300, H3K27me3, and H3K36me3. **(a)** All eccDNA shown in gray tracks. **(b)** Small eccDNA (1-5kb) shown in red tracks. **(c)** Medium eccDNA (5-20kb) shown in blue tracks. **(d)** Large eccDNA (>20kb) shown in purple tracks. Each row represents one size category. Vertical dashed lines mark eccDNA boundaries (eSS, eccDNA start site; eES, eccDNA end site). X-axis represents relative position across eccDNA regions; y-axis shows normalized signal intensity (RPM). Sample sizes indicated in individual panels. Stronger smoothing applied for visual clarity. **(e)** Quality control tests for purified circular DNA were performed using a qPCR assay.

Following CRISPR-Cas9 and Exonuclease V treatments, the levels of linear DNA and mtDNA in the total DNA sample were reduced. The circular p4339 plasmid, used as an internal control, remained detectable after the treatments. Ct, cycle threshold; p4339, inner control plasmid; mtDNA, mitochondrial DNA.

S1: Clinical characteristics of CRC cohort

ID	Tumour tissue samples	Normal tissue samples	Age	Sex	Resectability	Radical	Stage	Tumor size	Tumor location	Differentiation	Histology	Metastases code	Metastasis sites	Recurrence	Days to Recurrence	OS Event	Days to OS Event
CRC-021	CRCtx 021	CRCnx 021	64	2	1	R0	2a	3,5	8	9	1	0	0	0	2305	0	2305
CRC-022	CRCtx 022	CRCnx 022	73	2	1	R0	2a	8	1	1	1	0	0	0	2717	0	2717
CRC-023	CRCtx 023	CRCnx 023	79	2	1	R0	2a	4,5	1	9	1	0	0	0	2031	0	2031
CRC-024	CRCtx 024	CRCnx 024	81	2	1	R0	2a	10	3	9	1	0	0	0	1419	0	1419
CRC-025	CRCtx 025	CRCnx 025	50	1	1	R0	2b	3,5	9	9	1	0	0	1	876	0	2008
CRC-026	CRCtx 026	CRCnx 026	64	2	1	R0	2b	5,5	9	9	1	0	0	0	2613	0	2613
CRC-027	CRCtx 027	CRCnx 027	66	1	1	R0	3a	1,7	9	9	1	0	0	0	1639	0	1639
CRC-028	CRCtx 028	CRCnx 028	68	1	1	R0	3a	2,2	8	9	1	0	0	0	1615	0	1615
CRC-0	CRCtx 029	CRCnx 029	53	2	1	R0	3a	2	8	1	1	0	0	0	2094	0	2094

29																	
CRC-030	CRCtx 030	CRCnx 030	75	2	1	R0	3a	2,2	3	9	1	0	0	0	2256	0	2256
CRC-031	CRCtx 031	CRCnx 031	54	1	1	R0	3b	5,5	4	1	1	0	0	0	2668	0	2668
CRC-032	CRCtx 032	CRCnx 032	61	1	1	R0	3b	1,5	10	9	1	0	0	0	1972	0	1972
CRC-033	CRCtx 033	CRCnx 033	63	1	1	R0	3b	7	8	9	1	0	0	0	2032	0	2032
CRC-034	CRCtx 034	CRCnx 034	71	1	1	R0	3b	7	8	9	1	0	0	0	1659	0	1659
CRC-035	CRCtx 035	CRCnx 035	75	1	1	R1	3b	2	8	9	1	0	0	0	2409	0	2409
CRC-036	CRCtx 036	CRCnx 036	80	1	1	R0	3b	5	3	9	1	0	0	1	709	0	1955
CRC-037	CRCtx 037	CRCnx 037	66	2	1	R0	3b	1,5	6	1	1	0	0	0	1617	0	1617
CRC-039	CRCtx 039	CRCnx 039	75	2	1	R0	3b	5	9	1	1	0	0	0	2030	0	2030
CRC-	CRCtx 040	CRCnx 040	80	2	1	R0	3b	4,5	4	9	1	0	0	1	125	1	2513

040																	
CRC-046	CRCtx 046	CRCnx 046	51	1	1	R1	3b	7	8	9	2	0	0	0	2819	0	2819
CRC-047	CRCtx 047	CRCnx 047	67	2	1	R0	3b	2,3	9	2	1	0	0	1	378	0	2786
CRC-050	CRCtx 050	CRCnx 050	50	1	1	R0	3a	2	1	1	3	0	0	0	2491	0	2491
CRC-053	CRCtx 053	CRCnx 053	71	2	1	R0	3a	3	8	9	1	0	0	0	1987	0	1987
CRC-054	CRCtx 054	CRCnx 054	50	2	1	R0	3a	2	8	9	1	0	0	0	2626	0	2626
CRC-055	CRCtx 055	CRCnx 055	64	2	1	R0	3a	2,5	5	9	1	0	0	0	2354	0	2354

**KBA ID**		text		
**Frozen Tumor**				
**Frozen Normal**				
**Diagnosis**		1: colorectal cancer		
**Age**	Age at time of inclusion	number [years]		
**Sex**		1: Male	2: Female	
**Resectability**		0: No Operation 1: Operated 2: Opertion tried but was not possible		
**Radical**		text		
**Stage**		1-3b		
**Tumor location**		1: Cecum	2: Appendix	3: Ascending
**Histology**		1: adenocarcinoma	2: mucinouis	3: signet ring cell

**Recurrence**	recurrence	0: no 1: yes		
**Days to Recurrence**	days from blood sample to recurrence or progressive disease	number [count]		
**OS Event**		0: alive 1: dead		
**Days to OS Event**	days from blood sample to dead/follow up	number [count]		

**Characteristic**	Low eccDNA (n=12)	High eccDNA (n=12)	P-value	Statistical Test
**Demographics**				
**Age, years (median, IQR)**	67.0 (59.2-73.5)	66.5 (61.2-75.0)	0.98	Mann-Whitney U
**Sex, female n (%)**	5 (41.7%)	8 (66.7%)	0.41	Fisher's exact
**BMI, kg/m² (median, IQR)**	27.8 (25.0-29.2)	25.1 (22.4-26.7)	0.08	Mann-Whitney U
**Clinical Parameters**				
**Stage, n (%)**			0.46	Chi-square
**2a**	3 (25.0%)	1 (8.3%)		
**2b**	0 (0.0%)	1 (8.3%)		
**3a**	3 (25.0%)	5 (41.7%)		
**3b**	6 (50.0%)	5 (41.7%)		
**Tumor size, cm (median, IQR)**	5.0 (2.2-7.0)	2.8 (2.0-3.8)	0.26	Mann-Whitney U
**CACI score (median, IQR)**	2.0 (1.8-3.0)	2.5 (1.8-3.0)	0.86	Mann-Whitney U
**ASA score (median, IQR)**	1.0 (1.0-2.0)	2.0 (1.0-2.0)	0.24	Mann-Whitney U
**Diabetes, n (%)**	1 (8.3%)	1 (8.3%)	1	Fisher's exact

S2: No. of eccDNA-borne gene counts in TT, NAT and synthetic dataset. Permutation test results

Sample ID	TT	TT_ID	NAT	NAT_ID	Synthetic_data_mean	p-value
CRC-021	11486	CRCtx021	1101	CRCnx021	19775.84	0.0402
CRC-022	2811	CRCtx022	0	CRCnx022	2898.28	NA
CRC-023	4004	CRCtx023	1018	CRCnx023	2641.24	0.0339
CRC-024	2738	CRCtx024	22	CRCnx024	2163.44	0.1102
CRC-025	4083	CRCtx025	714	CRCnx025	2447.08	0.0384
CRC-026	0	CRCtx026	1640	CRCnx026	NA	0.04
CRC-027	13115	CRCtx027	1555	CRCnx027	20039.88	0.0349

CRC-028	11450	CRCtx028	1024	CRCnx028	18213.04	0.0388
CRC-029	3178	CRCtx029	2415	CRCnx029	2316.72	0.0356
CRC-030	9584	CRCtx030	185	CRCnx030	15131.48	0.0401
CRC-031	608	CRCtx031	334	CRCnx031	414.04	0.5388
CRC-032	3862	CRCtx032	1676	CRCnx032	3706.88	0.0351
CRC-033	10870	CRCtx033	583	CRCnx033	18403.12	0.5499
CRC-034	3446	CRCtx034	660	CRCnx034	3054.52	0.036
CRC-035	12100	CRCtx035	7312	CRCnx035	18614.08	0.0395
CRC-036	12045	CRCtx036	735	CRCnx036	20535.08	0.0373
CRC-037	9073	CRCtx037	1067	CRCnx037	14329.72	0.0427
CRC-039	4840	CRCtx039	905	CRCnx039	4081.16	0.0391
CRC-040	3926	CRCtx040	200	CRCnx040	2372.68	0.0384
CRC-046	2326	CRCtx046	736	CRCnx046	1694.08	0.0335
CRC-047	9431	CRCtx047	1022	CRCnx047	17294.36	0.0374
CRC-050	1945	CRCtx050	63	CRCnx050	1106	0.04
CRC-053	3301	CRCtx053	651	CRCnx053	2264.52	0.0354
CRC-054	10559	CRCtx054	795	CRCnx054	15366.2	0.0407
CRC-055	4917	CRCtx055	2557	CRCnx055	3650.28	0.8848

S3: Binomial test results

```
Total_count = 27684 # no. of genes in RNA expression matrix considered in kallisto hg38
n_total_genes = len(df2)  # Total DEGs in eccDNA
n_cancer_related =  len(onecc) # Cancer-related DEGs in eccDNA
expected_proportion = Total_count/len(onco)  # Proportion of cancer-related genes in the genome (as an example, 

print (n_cancer_related, n_total_genes, expected_proportion)
# Perform binomial test (one-sided)
print (stats.binom_test(n_cancer_related, n_total_genes, expected_proportion, alternative='greater').sum)
223 1480 9.99783315276273
```

S4: GSEA results

Gene Set Name	# Genes in Gene Set (K)	Description	# Genes in Overlap (k)	k/K	p	FDR q-value
HALLMARK_EPITHELIAL_MESENCHYMAL_TRANSITION	200	Genes defining epithelial-mesenchymal transition, as in wound healing,	18	0.09	3.95E-12	1.98E-10

		fibrosis and metastasis.				
HALLMARK_KRAS_SIGNALING_UP	200	Genes up-regulated by KRAS activation.	16	0.08	3.61E-10	9.03E-09
HALLMARK_ESTROGEN_RESPONSE_LATE	200	Genes defining late response to estrogen.	11	0.055	8.22E-06	1.37E-04
HALLMARK_COAGULATION	138	Genes encoding components of blood coagulation system; also up-regulated in platelets.	9	0.0652	1.41E-05	1.77E-04
HALLMARK_KRAS_SIGNALING_DN	200	Genes down-regulated by KRAS activation.	8	0.04	1.17E-03	1.17E-02
HALLMARK_ANDROGEN_RESPONSE	101	Genes defining response to androgens.	5	0.0495	3.98E-03	2.44E-02
HALLMARK_ESTROGEN_RESPONSE_EARLY	200	Genes defining early response to estrogen.	7	0.035	4.88E-03	2.44E-02
HALLMARK_MYOGENESIS	200	Genes involved in development of skeletal muscle (myogenesis).	7	0.035	4.88E-03	2.44E-02
HALLMARK_TNFA_SIGNALING_VIA_NFKB	200	Genes regulated by NF-kB in response to TNF [GeneID=7124].	7	0.035	4.88E-03	2.44E-02
HALLMARK_XENOBIOTIC_METABOLISM	200	Genes encoding proteins involved in processing of drugs and other xenobiotics.	7	0.035	4.88E-03	2.44E-02
HALLMARK_PANCREAS_BETA_CELLS	40	Genes specifically up-regulated in pancreatic beta cells.	3	0.075	8.06E-03	3.66E-02

S5: ENCODE accession numbers for ChIP and ATAC data

Target of assay	Biosample term name	Biosample age	BigWig file	Bed file
ATAC-seq	transverse colon	37 years	ENCFF469WCM	ENCFF054QTC
CTCF	transverse colon	37 years	ENCFF816TDF	ENCFF681RYK
EP300	transverse colon	37 years	ENCFF093NJN	ENCFF754LGO
H3K27ac	transverse colon	37 years	ENCFF959PXE	ENCFF142COP
H3K27me3	transverse colon	37 years	ENCFF612HRT	ENCFF411ZRA
H3K36me3	transverse colon	37 years	ENCFF090EKJ	ENCFF990VOU
H3K4me1	transverse colon	37 years	ENCFF997VIZ	ENCFF693QVL
H3K4me3	transverse colon	37 years	ENCFF219ZIO	ENCFF799XQH
H3K9me3	transverse colon	37 years	ENCFF150JBJ	ENCFF885GZB
POLR2A	transverse colon	37 years	ENCFF976VVU	ENCFF838LKZ

S6: Chromatin enrichment analysis

mark	analysis_type	mean_fold_enrichment	se_fold_enrichment	ci_95_lower	ci_95_upper	pvalue	pvalue_corrected	cohens_d	significant_corrected
EP300	all	1.017	0.001	1.014	1.019	1.19E-07	2.27E-07	2.72	TRUE
EP300	small	1.018	0.001	1.015	1.020	1.19E-07	2.27E-07	2.87	TRUE
EP300	medium	1.008	0.001	1.006	1.010	1.19E-07	2.27E-07	1.60	TRUE
EP300	large	0.991	0.003	0.985	0.998	0.012605	0.014005	-0.59	TRUE
ATAC	all	1.057	0.005	1.046	1.068	1.19E-07	2.27E-07	2.31	TRUE
ATAC	small	1.063	0.005	1.053	1.073	1.19E-07	2.27E-07	2.76	TRUE
ATAC	medium	1.023	0.006	1.009	1.036	0.002001	0.002425	0.74	TRUE
ATAC	large	0.855	0.016	0.822	0.888	1.19E-07	2.27E-07	-1.91	TRUE
POLR2A	all	1.031	0.002	1.027	1.034	1.19E-07	2.27E-07	3.60	TRUE
POLR2A	small	1.035	0.002	1.032	1.038	1.19E-07	2.27E-07	4.65	TRUE
POLR2A	medium	1.020	0.002	1.016	1.024	1.19E-07	2.27E-07	2.13	TRUE
POLR2A	large	0.858	0.022	0.813	0.904	2.38E-07	4.15E-07	-1.34	TRUE
H3K27ac	all	1.017	0.005	1.007	1.028	0.001238	0.001597	0.72	TRUE
H3K27ac	small	1.030	0.002	1.026	1.034	1.19E-07	2.27E-07	3.03	TRUE
H3K27ac	medium	1.035	0.004	1.027	1.044	1.19E-07	2.27E-07	1.79	TRUE
H3K27ac	large	0.668	0.041	0.583	0.753	1.19E-07	2.27E-07	-1.69	TRUE
H3K4me1	all	0.990	0.022	0.944	1.035	0.252249	0.265526	-0.10	FALSE
H3K4me1	small	1.040	0.004	1.032	1.048	1.19E-07	2.27E-07	2.12	TRUE
H3K4me1	medium	1.043	0.006	1.030	1.055	1.19E-07	2.27E-07	1.51	TRUE
H3K4me1	large	0.765	0.037	0.688	0.843	4.77E-07	7.63E-07	-1.38	TRUE
H3K9me3	all	0.993	0.006	0.981	1.005	0.583806	0.583806	-0.26	FALSE
H3K9me3	small	1.005	0.001	1.004	1.007	1.67E-06	2.57E-06	1.24	TRUE
H3K9me3	medium	1.011	0.003	1.005	1.017	0.0014	0.00175	0.76	TRUE
H3K9me3	large	0.873	0.043	0.785	0.961	2.26E-06	3.36E-06	-0.62	TRUE
CTCF	all	1.046	0.005	1.036	1.055	1.19E-07	2.27E-07	2.09	TRUE

CTCF	small	1.049	0.005	1.039	1.058	1.19E-07	2.27E-07	2.24	TRUE
CTCF	medium	1.024	0.003	1.017	1.031	2.38E-07	4.15E-07	1.55	TRUE
CTCF	large	0.894	0.013	0.868	0.921	1.19E-07	2.27E-07	-1.72	TRUE
H3K36me3	all	0.987	0.005	0.976	0.997	0.009576	0.010944	-0.54	TRUE
H3K36me3	small	0.999	0.001	0.997	1.001	0.406112	0.416525	-0.17	FALSE
H3K36me3	medium	1.008	0.002	1.003	1.012	0.005898	0.006938	0.69	TRUE
H3K36me3	large	0.883	0.041	0.799	0.968	0.000239	0.00033	-0.59	TRUE
H3K4me3	all	1.021	0.002	1.018	1.025	1.19E-07	2.27E-07	2.88	TRUE
H3K4me3	small	1.026	0.001	1.023	1.028	1.19E-07	2.27E-07	4.07	TRUE
H3K4me3	medium	1.024	0.002	1.020	1.029	1.19E-07	2.27E-07	2.46	TRUE
H3K4me3	large	0.715	0.027	0.658	0.771	1.19E-07	2.27E-07	-2.17	TRUE
H3K27me3	all	0.966	0.014	0.936	0.996	0.187501	0.202704	-0.49	FALSE
H3K27me3	small	1.004	0.001	1.002	1.005	8.34E-06	1.19E-05	1.16	TRUE
H3K27me3	medium	1.007	0.002	1.003	1.010	0.000742	0.00099	0.79	TRUE
H3K27me3	large	0.849	0.041	0.764	0.934	4.77E-07	7.63E-07	-0.81	TRUE

S7: Description of primers for linear DNA fragment synthesis, qPCR and PCR reactions.

Gene	Primer sequence	Fragment length
GNP1 linear spike-in	F - GGTTCAAAGGTGTCGTTGCC	337 bp
	R - GCACCGTTAGCAACGGAAAG	
AGP1 linear spike-in	F - GTTTTGGGTTTGCAGTCGCT	820 bp
	R - GCACAGAAGGCAATAACGGC	
ACT1 linear spike-in	F - TGGATTCTGGTATGTTCTAGC	1409 bp
	R - GAACGACGTGAGTAACACC	
BCP1 linear spike-in	F - TCAGTACAGTTGCGGTGGAC	2716 bp
	R - TCGGATAGCCTCTGGTTAGG	
qPCR for mtDNA	F - GCCCACTTCCACTATGTCCT	92 bp
	R GATTTTGGCGTAGGTTTGGTCT	
qPCR for BCP1	F - CGGTGGTAACCCAGAAGTTGA	130 bp
	R - TGTGGTGGTTGGGGAACCTA	
qPCR p4339	F - TGCCCTGCCCCTAATCAGTA	60 bp
	R - CTGGGCAGATGATGTCGAGG	
qPCR CXCL5 gene	F - TGACAAGGGTGGAGTAGAAAGG	133 bp
	R - TCTCTGCTGAAGACTGGGAAAC	
qPCR ALB	F - TGAAACATACGTTCCCAAAGAGTTT	81 bp

	R - CTCTCCTTCTCAGAAAGTGTGCATAT	
PCR Cox5B	F - GGGCACCATTTTCCTTGATCAT	119 bp
	R - AGTCGCCTGCTCTTCATCAG	
[CXCL5^{circle}]	F - AGCTGCTGGCAAGGATCAG	737 bp
	R - ATTTGTGTGGCATTGTAGGAAGC	
